# Microbial strain-level population structure and genetic diversity from metagenomes

**DOI:** 10.1101/gr.216242.116

**Published:** 2017-04

**Authors:** Duy Tin Truong, Adrian Tett, Edoardo Pasolli, Curtis Huttenhower, Nicola Segata

**Affiliations:** 1 Centre for Integrative Biology, University of Trento, 38123 Trento, Italy;; 2 Biostatistics Department, Harvard School of Public Health, Boston, Massachusetts 02115, USA;; 3 The Broad Institute, Cambridge, Massachusetts 02142, USA

## Abstract

Among the human health conditions linked to microbial communities, phenotypes are often associated with only a subset of strains within causal microbial groups. Although it has been critical for decades in microbial physiology to characterize individual strains, this has been challenging when using culture-independent high-throughput metagenomics. We introduce StrainPhlAn, a novel metagenomic strain identification approach, and apply it to characterize the genetic structure of thousands of strains from more than 125 species in more than 1500 gut metagenomes drawn from populations spanning North and South American, European, Asian, and African countries. The method relies on per-sample dominant sequence variant reconstruction within species-specific marker genes. It identified primarily subject-specific strain variants (<5% inter-subject strain sharing), and we determined that a single strain typically dominated each species and was retained over time (for >70% of species). Microbial population structure was correlated in several distinct ways with the geographic structure of the host population. In some cases, discrete subspecies (e.g., for *Eubacterium rectale* and *Prevotella copri*) or continuous microbial genetic variations (e.g., for *Faecalibacterium prausnitzii*) were associated with geographically distinct human populations, whereas few strains occurred in multiple unrelated cohorts. We further estimated the genetic variability of gut microbes, with *Bacteroides* species appearing remarkably consistent (0.45% median number of nucleotide variants between strains), whereas *P. copri* was among the most plastic gut colonizers. We thus characterize here the population genetics of previously inaccessible intestinal microbes, providing a comprehensive strain-level genetic overview of the gut microbial diversity.

Strain-level variants within microbial species are crucial in determining their functional capacities within the human microbiome, including interaction with host tissues (Bron et al. 2012), modulation of immune homeostasis (Needham et al. 2013), and xenobiotic metabolism (Spanogiannopoulos et al. 2016). Pathogenic potential is also strain-specific in many species, including *Escherichia coli*, which is prevalent in the healthy human gut despite some strains causing life-threatening infections (Bielaszewska et al. 2011; Loman et al. 2013) or mucosal necrosis in premature infants (Ward et al. 2016). Strain-level microbial genomic variation typically consists of single-nucleotide variants (SNVs) as well as acquisition/loss of genomic elements including genes, operons, or plasmids (Tettelin et al. 2005). Although these genomic features can be accurately characterized in microbial isolates, they have been difficult to study using culture-independent approaches, despite thousands of human-associated metagenomes being available. Translational applications of the human microbiome will require analysis of each community's microbial strain population, ideally in high-throughput from culture-independent sequencing.

Advances in metagenome bioinformatics over the last decade have refined the resolution of microbial community taxonomic profiling from the phylum to the species, but it is still difficult to characterize microbes in communities at the strain level. Metagenomic assembly (Nagarajan and Pop 2013) provides one solution and has been successful in identifying strains of uncharacterized species (Narasingarao et al. 2012; Brown et al. 2015). However, compared to assembling single isolates, metagenomic assembly is computationally challenging, both in efficiency and methodologically in addressing fragmentary contigs, binning, and avoiding chimeric assemblies that combine multiple related strains. To improve metagenomic assembly, extensions that coassemble multiple metagenomes are also available (Alneberg et al. 2014; Imelfort et al. 2014), but accurate assemblies can require time-consuming manual curation (Sharon et al. 2013; Raveh-Sadka et al. 2015), and it is difficult to generalize the approach to large sets of metagenomes and low abundance microbes.

For microbial communities such as the human microbiome supported by sufficient isolate reference sequences, it is alternatively possible to map the reads of a metagenome against reference genomes and obtain a survey of the single-nucleotide variant (SNV) patterns across samples (Schloissnig et al. 2013). Recent signature-based approaches based on marker genes (Franzosa et al. 2015; Luo et al. 2015; Truong et al. 2015) or pan-genes (Scholz et al. 2016) are also able to identify and track strains across samples, but they do not typically allow comprehensive strain cataloging among metagenomes or the reconstruction of microbial phylogenetic relationships in a manner comparable to studies of isolate genomes. As such, it has remained difficult, or in many cases, impossible, to profile strains from metagenomes and compare them across a large set of microbiome samples with the same level of resolution attainable by isolate comparative genomics.

In this work, we present StrainPhlAn, a novel method and implementation to profile microbial strains from metagenomes at a resolution comparable with that of isolate sequencing and apply it to thousands of gut samples spanning multiple host populations. The method is based on reconstructing consensus sequence variants within species-specific marker genes and using them to estimate strain-level phylogenies. StrainPhlAn allowed us to process >7 TB of sequencing data from the largest available metagenomic investigations (Qin et al. 2010, 2012; Human Microbiome Consortium 2012; Karlsson et al. 2013; Le Chatelier et al. 2013; Nielsen et al. 2014; Zeller et al. 2014; Obregon-Tito et al. 2015; Rampelli et al. 2015), yielding large-scale strain-level phylogenies that are used to study the population genomics, biogeography, genetic diversity, and strain retention for 125 intestinal species, most of which are sparsely represented in current culture-based investigations.

## Results

### Enabling strain-level meta-analytic epidemiology of microbial communities and the human microbiome

We developed a novel computational approach to study the strain-level genetics of microbes directly from metagenomic samples and to infer the phylogenetic structure of species across samples. Strains are profiled in each sample by reconstructing a sufficient subset of their genomes for variant calling, which provides a nucleotide-level consensus sequence for each strain. This is carried out by mapping metagenomic reads against species-specific marker sequences (up to 200 per species from a total set of ∼1 million markers) that are broadly conserved within each species and do not have substantial sequence similarity with genomic regions in other species (Truong et al. 2015). This approach allows strain-specific consensus sequence identifications from as few as a single reference genome (Methods; Supplemental Fig. S1), and we have verified that variants in these marker genes are representative of whole-genome variability (Supplemental Figs. S2, S3). The reconstructed strain-specific consensus is independent from the sequence of the marker used as a backbone for the mapping and can be used for standard phylogenetic analysis, typically multiple sequence alignment (Maiden et al. 1998), followed by phylogenetic reconstruction (Stamatakis 2014). They can also be used to infer the population structure of strains directly from sets of metagenomes, similarly to the analysis of isolate genomes using shared (core) genes for phylogenetic, population biology, and comparative genomics (Budroni et al. 2011; Segata et al. 2013; Page et al. 2015).

**Figure 1.**
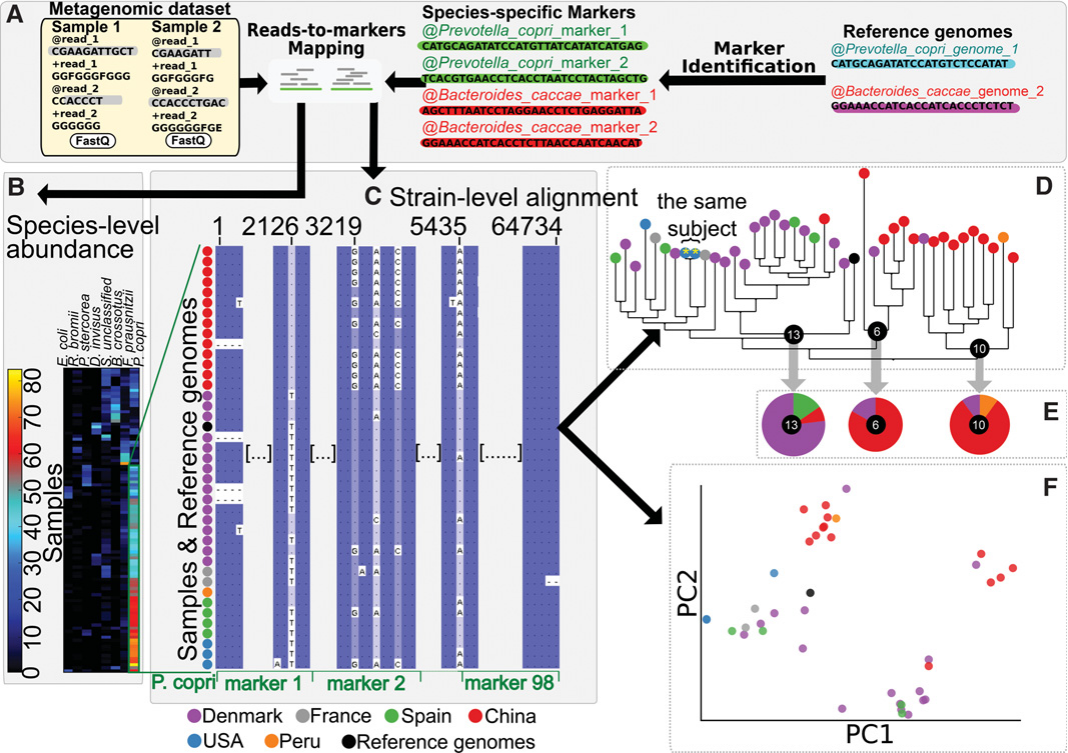
StrainPhlAn for strain identification and tracking in shotgun metagenomes and its application to *Prevotella copri* in the human gut. StrainPhlAn provides a method to identify strains from shotgun metagenomes and provides tracking, comparative, and phylogenetic analyses across samples. Here, we illustrate results using *Prevotella copri* as an example species in a demonstration subset of this study's human gut metagenomes. (*A*) In this overview of the method, for each species for which strains are to be analyzed across a metagenome collection, sample-specific and strain-specific markers are constructed by mapping reads against the MetaPhlAn2 (Truong et al. 2015) database of species-specific reference sequences. (*B*) In each sample, species are identified and quantified if sufficient coverage for the species markers is detected. Here, 100 samples with sufficiently abundant *P. copri* are shown (seven other abundant species are also displayed). (*C*) The preselected species-specific markers are concatenated, aligned, and variants identified using the consensus sequence of mapped metagenomic reads. (*D*) From the resulting set of the most abundant strains per sample, a phylogenetic tree can be built. This allows, for example, retained or minimally divergent strains within a particular environment (e.g., human host) to be easily identified when they appear within the same subtrees. (*E*) Strains or subtrees can also be statistically associated with sample metadata (e.g., human or environmental phenotypes). (*F*) Each species’ genetic diversity and divergence can be easily visualized as an ordination comparable to those used for isolate or human population genetics.

*Prevotella copri* is a key example of a human commensal for which strain-level comparative genomics from metagenomes is particularly important, because only one cultured isolate reference genome is currently available (Hayashi et al. 2007). It is a frequent colonizer of the human gut (Qin et al. 2010; Human Microbiome Consortium 2012) that, unusually, occurs abundantly (Arumugam et al. 2011) in only a fraction of the population (from 10% to 25%) (Koren et al. 2013) and has been strongly associated with the onset of rheumatoid arthritis (Scher et al. 2013; Zhang et al. 2015). It is also difficult to culture ex vivo, leading to very limited phenotypic and genotypic characterization (Hayashi et al. 2007). By applying StrainPhlAn on *P. copri* directly from gut metagenomes (Fig. 1), we can provide the first characterization of its population genomics in three complementary ways.

First, StrainPhlAn provides a strain-level phylogeny of each analyzed species (in this instance, *P. copri*) from the concatenated alignment of the markers (Fig. 1B,C). When metagenomes are accompanied by phenotypic, environmental, or other metadata annotations, these can be tested for significant association relatively to the population genomic structure of *P. copri* within one or more subclades of the phylogeny (Fig. 1D). Finally, the population structure can be visualized by ordination to further identify substructures (e.g., subspecies) in the genetic diversity of the strains (Fig. 1E). Strain-specific consensus sequences from available reference genomes (again, notably only one for *P. copri*) can be included in any of these analyses to compare culture-based genomic information with that extracted from the metagenomes.

Even using this limited set of samples, one can already identify several key new features of *P. copri* population biology. Essentially identical strains are carried in the two longitudinal samples from the same subject (Fig. 1D), and a diverged subspecies clade is also carried almost exclusively within the Chinese population, which is one of roughly four subspecies-level clades of related strains. Even with the reduced set of samples shown here for illustration (for the complete analysis, see Fig. 4C below), this analysis highlights the use of StrainPhlAn for population genomics of phenotype-relevant microbial community members who are recalcitrant to culture-based approaches.

### StrainPhlAn achieves per-nucleotide error rates <0.1%

We first validated the method's precision at the per-nucleotide level using two metagenomes from the HMP mock community (Human Microbiome Consortium 2012) comprising 21 known organisms (Methods). This resulted in an error rate (fraction of incorrect nucleotides) <0.05% overall (Supplemental Table S1). This performance was confirmed on 36 synthetic data sets containing multiple strains from the same species (Methods) in which we achieved even lower error rates (<0.03%) for species with coverage greater than 2× (Supplemental Fig. S4), and when considering 36 additional semisynthetic data comprising gut metagenomes spiked with in silico strain-specific reads (<0.2% error rate) (Supplemental Fig. S4). When compared to other recent strain-level metagenomic profilers, StrainPhlAn achieved substantially better results than MIDAS (Nayfach et al. 2016) and ConStrains (Luo et al. 2015), based on per-nucleotide and overall strain-tracking accuracies, respectively (Supplemental Tables S1–S5; Supplemental Figs. S5, S6). In this evaluation, StrainPhlAn was the only method to achieve a resolution in culture-independent strain reconstruction that is comparable with that of isolate genome analysis, which is necessary for accurate phylogenetic reconstruction (Supplemental Figs. S5, S6).

We further validated the accuracy of strain identification in vivo by using previously sequenced stool samples (Nielsen et al. 2014) from subjects sampled after the intake of a known commercial probiotic bacteria, specifically *Bifidobacterium animalis* subsp. *lactis* (strain *CNCM I-2494*). In the original work (Nielsen et al. 2014), reconstruction of the *B. animalis* strain was performed by merging together 19 metagenomes from subjects challenged with the probiotic and analyzing the pooled reads. Importantly, this is only possible in cases in which it is known a priori that the same strain will appear in multiple samples; so the method does not generalize well to most microbes and samples. In contrast, StrainPhlAn allows the analysis of any strain with sufficient sequencing depth per sample, and here we targeted the seven samples in which the markers of the *B. animalis* species recruited at least 2× coverage. Comparison of our inferred strain consensus profiles to the reference genome achieved <0.01% single nucleotide errors, which is two orders of magnitude lower than the average nucleotide variation (1.3%) between strains from isolate sequencing in the *B. animalis* species and again one or more orders of magnitude lower than the error rate produced by MIDAS (Supplemental Table S6). The phylogeny built by StrainPhlAn using these sequences further placed the *B. animalis* found in these samples among the cluster of reference genomes for this probiotic organism that has been sequenced and assembled several times independently (Supplemental Figs. S7, S8; Supplemental Table S7). Our approach is also computationally efficient; this example on real gut metagenomes required ∼20 min per sample and can be further accelerated by parallelization or distributed computing (Methods), making it appropriate for hundreds of species spanning thousands of metagenomes.

### Integrated strain-level population genomics using more than 1500 human gut metagenomes

We next applied StrainPhlAn to a set of 1590 gut metagenomes from adult subjects retrieved from nine public data sets (Table 1) that we preprocessed using uniform quality control criteria (Methods) as in Pasolli et al. (2016). The resulting population spanned all continents except Australia and Antarctica, with curated common metadata including country of origin, health or disease state, age, and BMI (other metadata was either not provided or not common among data sets). It is important to consider that for strain-level population epidemiology, batch effects resulting from differences in sample collection, storage, DNA extraction, or library preparation are known to affect quantitative profiling, but they are unlikely to influence strain consensus sequence reconstruction from markers. All further subsequent analyses are thus performed on this large set of metagenomes that is diverse in its geographical location, human population of origin, and microbial genetic structure.

**Table 1.**
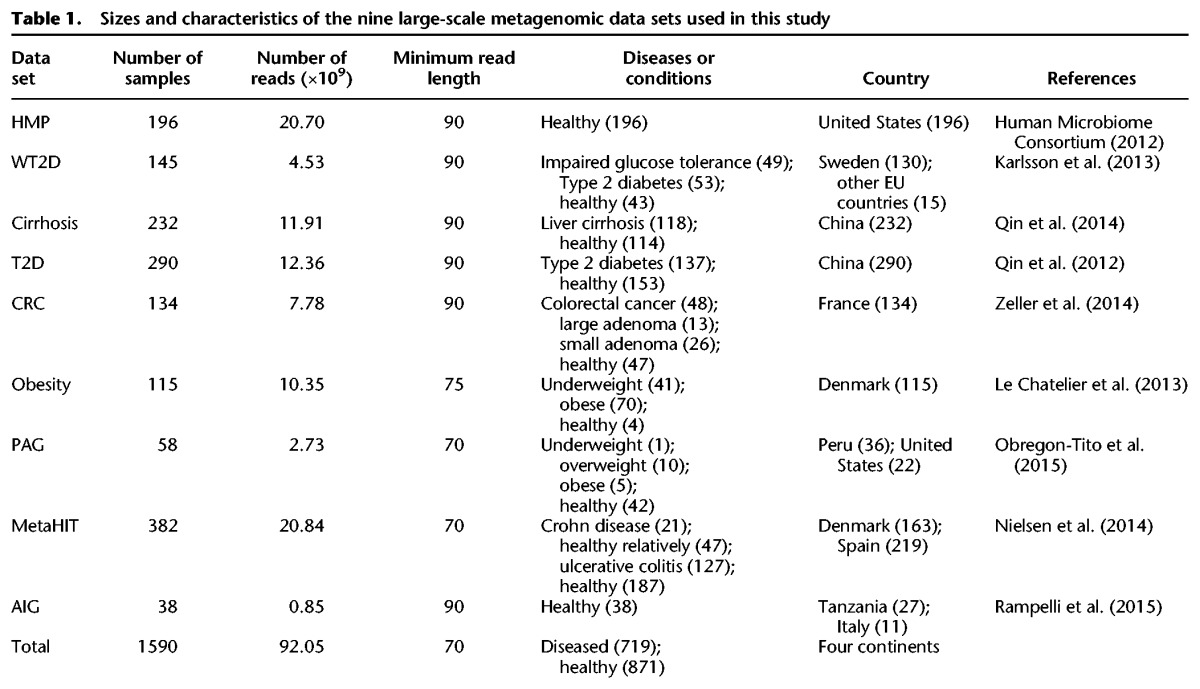
Sizes and characteristics of the nine large-scale metagenomic data sets used in this study

We note that despite a large body of work on strain-level phenotypic characterization and genetic comparisons from microbial isolates, a clear definition of the concept of “strain” is still lacking (Dijkshoorn et al. 2000; Konstantinidis et al. 2006). Genomes differing by just one or a few nucleotides could be defined as different strains, but such limited genetic differences may not result in any phenotypic changes (e.g., synonymous mutations) and would lead to the differentiation of strains in just a few microbial generations. Defining a broader genetic variation threshold can be effective in specific investigations, which is the approach taken by Operational Taxonomic Unit (OTU) definitions in amplicon profiling (Hamady and Knight 2009). However, such hard-limited sequence identity thresholds may be an oversimplification, because they are difficult to set universally and are both locus- and organism-specific. Phylogenetic modeling overcomes the need of defining hard cutoffs for strain or other clade boundaries, and we use this approach to estimate strain relatedness. However, when checking for strain identity is necessary, it is possible to set the threshold considering the intra-individual similarity of retained strains as compared to intra-individual strain heterogeneity.

### Single strains dominate most species in the gut microbiome

Analysis of microbial population structures was previously only possible using relatively laborious sequencing of isolate collections; here, we perform high-throughput strain-level profiling directly from a large set of metagenomes spanning multiple geographical locations. In contrast to the 73 prevalent species present in >50% of our more than 1500 samples, only 10 human-associated species can comparably take advantage of more than 750 sequenced isolates for comparative genetics, and of these, only *E. coli* is typically found in the gut. Moreover, sequencing isolates relies on cultivability, whereas our method can investigate members of the human microbiome (or other microbial communities) with fewer biases and no cultivation efforts.

**Figure 2.**
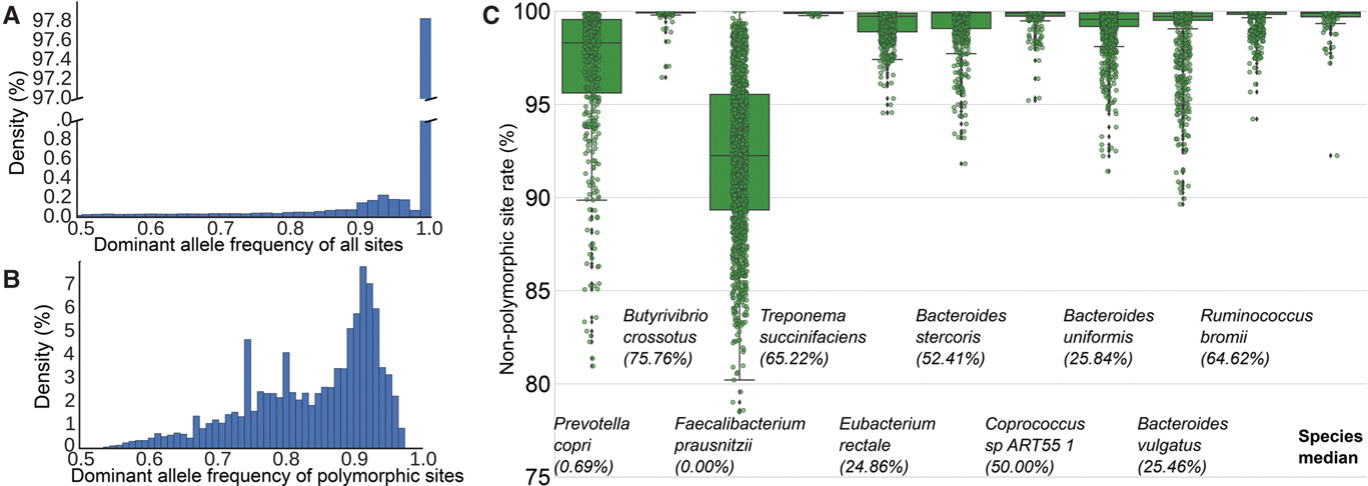
Most species are dominated by a single strain in the human gut. (*A*) Distribution of dominant allele frequency for all nucleotide positions in concatenated species-specific markers across all analyzed samples (>482 million total nucleotides). (*B*) Distribution of the dominant allele frequency for polymorphic positions. We report the median frequencies for each species/sample pair. (*C*) Distribution of nonpolymorphic site prevalence in samples for the 10 most prevalent gut bacterial species (for the full set of species, see Supplemental Fig. S9). The fraction of nonpolymorphic sites varies from sample to sample and from species to species. In parentheses, we quantify the percentage of strains with >99.9% of nonpolymorphic sites.

StrainPhlAn reconstructs each species’ most abundant strain per sample, and it can assess whether nondominant strains are present by identifying single nucleotide polymorphisms. We thus first validated the assumption that reconstructing each species’ most abundant strain per sample captures most strain-level diversity by assessing how frequently multiple strains per species are detectable in this sample set. In the human gut, most species were represented by a single dominant strain because they show <0.1% of nucleotides on the species-specific markers that are polymorphic. At this conservative threshold, for 35.7% of cases, no evidence of multiple strains was found at the given depth of sequencing (average 5.8 × 10^9^ nt/sample). Moreover, when the presence of more than one strain was detected, a single strain accounted for at least 80% of each species in another 44.4% of cases. This was determined by identifying, for all samples and markers, the polymorphic and nonpolymorphic sites (accounting for sequencing errors) (Methods) and by further estimating the allelic frequency of the dominant variant in polymorphic sites. In order to filter out potentially overestimated frequencies for the cases in which an allele is shared by more than one nondominant strain of a species in the sample, we consider the median of the frequencies of the dominant allele across polymorphic sites as the frequency of the dominant strain relative to the nondominant ones. The large majority (>97.8%) of marker nucleotides were not polymorphic at all (Fig. 2A), whereas the remaining nucleotides were largely dominated by a single variant.

Considering all cases in which a species is found in a sample (species-sample combinations), multiple strains were detectable in 14,698 cases (64.3% of species-sample combinations), but it was still rare to find two or more strains at comparable abundance (Fig. 2B). The dominant consensus sequence was less than twice as abundant as all others combined in only 5% of multistrain cases (Fig. 2B); in half of the cases, one strain dominated the others by at least 7:1 or more. Importantly, all these considerations are independent from the abundance of the species in the samples (Supplemental Fig. S10). When multiple strains were detectable, the fraction of samples in which they were detected varied considerably among species. *Butyrivibrio crossotus*, for example, did not show evidence of strain mixtures in 75% of the 156 samples in which it was detected, whereas more than one strain of *Faecalibacterium prausnitzii* were present in 100% of its 1052 samples (Fig. 2C). Even in these mixed species, a single strain typically dominated: The main strain of *P. copri* reached on average 86% relative abundance within the species, and similarly for *Butyrivibrio crossotus* (91%), *Faecalibacterium prausnitzii* (78%), *Bacteroides uniformis* (87%), *Bacteroides vulgatus* (91%), and *Ruminococcus bromii* (87%) (for a full list of species, see Supplemental Table S3).

Our analysis suggests that the ecology of multiple closely related strains in the human gut is characterized by the quantitative dominance of one strain. Given the roughly log-normal distribution of species abundances in microbiomes (Li et al. 2012) and the consequent long tail of low-abundance species, nondominant strains of a species are likely close to or below the limit of detection at typical sequencing depths. Even in an idealized case, a strain making up 5% of a species that itself represents 5% of the overall community, for example, requires a sequencing depth of ∼2 × 10^9^ nt to be detected reliably; any strains or species less abundant than this will not be detectable in a typical gut metagenome. Modeling these low-abundance, nondominant strains would thus largely result in low-quality phylogenetic information and would weaken the advantage of using species-specific marker genes. At the same time, this property makes it very accurate to rely on the frequency of the dominant allele, because potential alleles shared by nondominant strains can have only a minimal impact on the dominant-allele frequency. In our cross-sectional population genomic analysis, we thus focus on the dominant strain of each detected species, and StrainPhlAn also labels as potentially noisy the rare cases in which two strains from the same species are present at comparable abundances (Methods).

### Gut microbial stability and uniqueness are explained by subject-specific strain retention

With StrainPhlAn, we were able to explain previously observed community-level gut microbiome stability and individuality (Schloissnig et al. 2013; Franzosa et al. 2015) through a mechanism of within-subject strain retention. This parallels the assessment of within-subject strain retention that has been carried out previously for targeted pathogen isolates (Covacci et al. 1999; Nowrouzian et al. 2005; Bidmos et al. 2011). We estimated retention of strains in the gut microbiome by looking at multiple samples from the same participants available from the HMP (Human Microbiome Consortium 2012) in the absence of disease, and from MetaHIT (Qin et al. 2010; Nielsen et al. 2014), which includes 66 longitudinally sampled patients with inflammatory bowel disease (IBD). To this end, we defined our measure of genetic distance between strains as the length-normalized rate of single-nucleotide variants (SNVs) between the full set of markers considered in each species.

**Figure 3.**
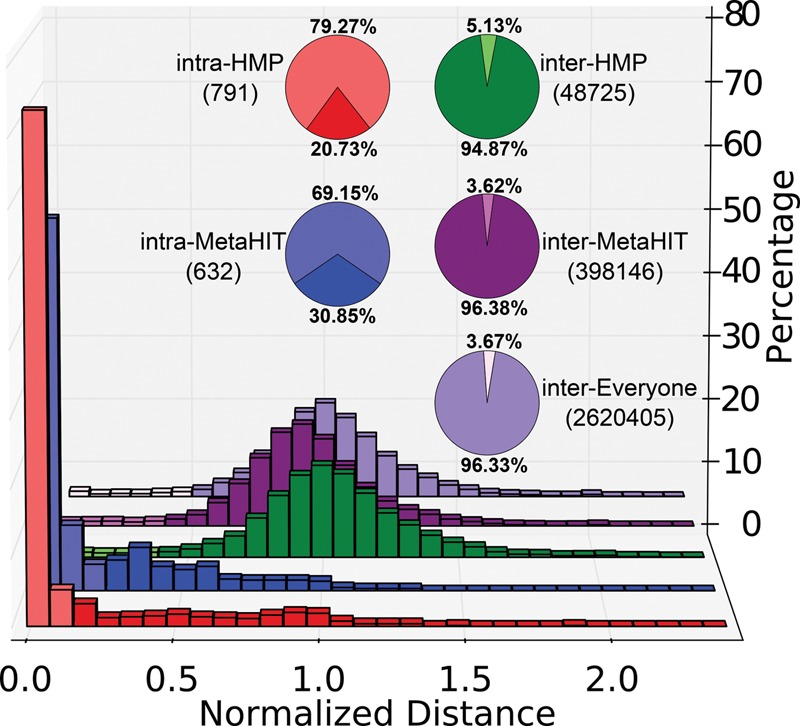
Most strains are retained over time within the human gut, but few strains are carried by multiple subjects. The distribution of the all-versus-all normalized genetic distance between strains is reported for increasingly large metagenome collections (only MetaHIT, only the HMP, or all 1590 samples). For MetaHIT and the HMP, we also computed the intra-subject distances (temporal separation between samplings averaging 163 SD 125 d and 219 SD 69 d, respectively) normalized based on the median of the all-versus-all comparisons.

We found that when looking at the same species in two samples from the same individual, the dominant strain of that species was exactly the same in 69% of the longitudinally sampled subjects in MetaHIT and 79% in those from the HMP (Fig. 3) with a percentage of 3.4% and 10.4% of strains that are lost or replaced, on average, each month in the two data sets (Supplemental Fig. S11). The fraction of shared species along longitudinal time points was lower (62.2% in the HMP and 61.1% in MetaHIT), suggesting that detectable species composition is slightly more dynamic than long-term strain retention. This could be explained, for example, by the hypothesis that species are rarely displaced by closely related competitors, or that when a strain of a species varies in abundance below the limit of detection, it may still be detected later as the same strain. These results help to explain why a strain-level signature of a subject's microbiome is constant in time, particularly in the absence of perturbations from the environment or disease (Franzosa et al. 2015).

In contrast with intra-subject strain retention, strains were rarely shared among individuals: We found evidence of the same strain shared between multiple individuals colonized by a common species in only 3.67% of cases (Fig. 3). A larger fraction of the population shared the same species (35.31% species in common, on average, between two different individuals). Shared geography did not increase the fraction of strains shared by different subjects, as it did not differ significantly within Europe (3.62%) versus worldwide (3.67%). Strains were slightly more commonly shared in the American samples of the HMP (5.13%), but species were less likely to be shared within the HMP (36.0%) compared with MetaHIT (40.5%). Both of these properties might vary on a less coarse geographical scale, however, and the population enrolled in the HMP was healthy as compared to MetaHIT's longitudinally sampled IBD patients, perhaps leading to greater strain diversity in the latter. Altogether, our analysis highlights the substantial longitudinal strain retention within the same microbial community and the relatively low proportion of strains shared between multiple individuals.

### Strain-level microbial genetics strongly correlate with geographically separated host populations

The evolution of specific host-associated microbes is closely linked to factors such as host migrations and transmission mechanisms (vertical, horizontal, environmental); for example, *Helicobacter pylori* is largely vertically transmitted (Delahay and Rugge 2012); as a result, its population genetics is closely linked to the ancestry and geography of its human hosts (Covacci et al. 1999; Suzuki et al. 2012). In this multicontinent meta-analysis, StrainPhlAn permitted the population structure of dominant strains of all species above the limit of detection to be determined in high-throughput. This enabled us to first assess which species comprised strains forming a continuum within the overall species diversity versus those with discrete clusters of strains forming subspecies clades (SCs). The former may be reflective of primarily horizontal transmission between hosts enabling freer gene flow, whereas the latter may reflect subspeciation due to primarily vertical transmission. In either case, the resulting microbial population structure can be further categorized as randomly or nonrandomly assorted geographically and with respect to host populations.

**Figure 4.**
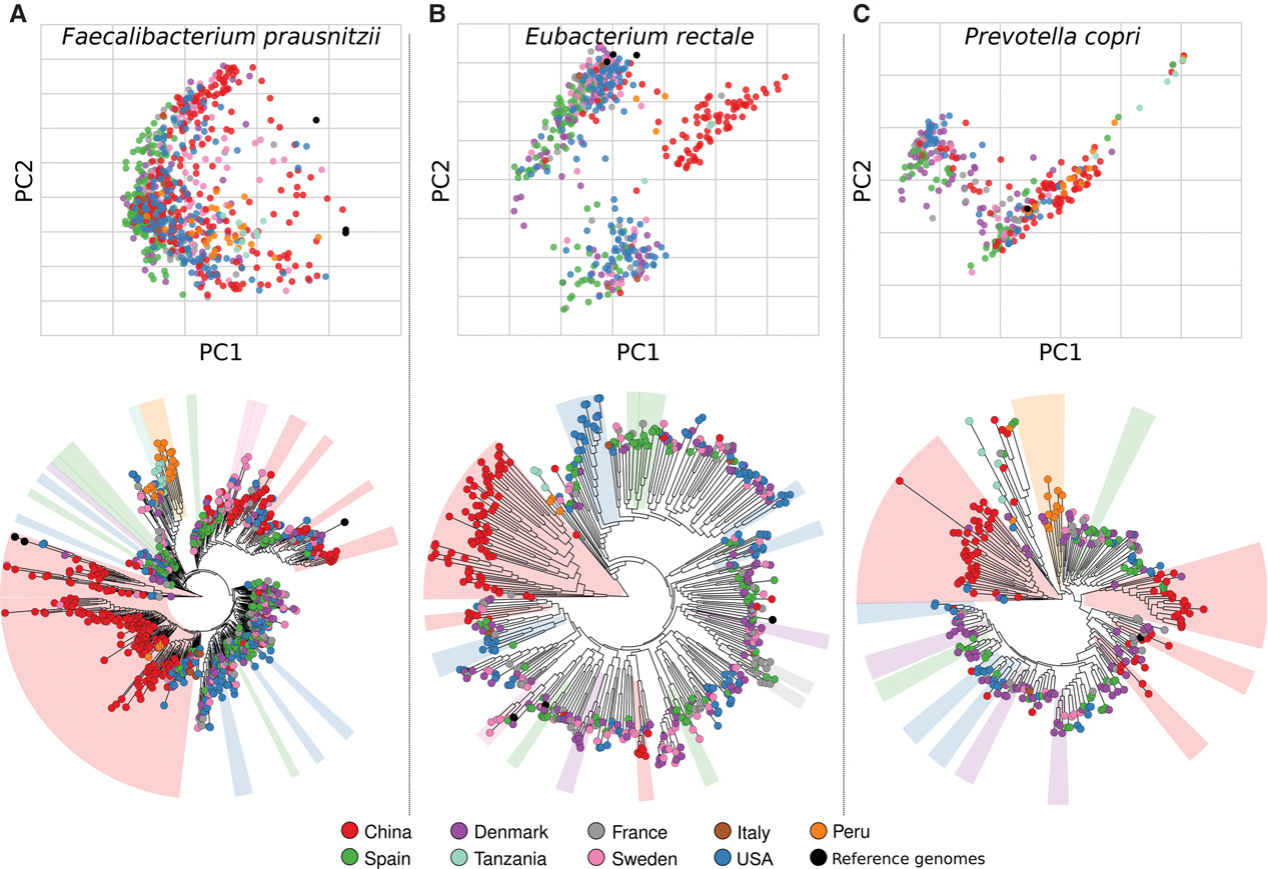
Population genetic structure of three common intestinal species and its association with sampling geography. Strain population structures for three representative human gut species, reported both as phylogenies built on the concatenated alignments of each species-specific reconstructed marker set (*bottom*). To highlight the presence of discrete clusters of related strains, we also report the genetic distances measured on the alignments as principal coordinate ordinations (*top*). We report the population structure of *Faecalibacterium prausnitzii* (*A*), *Eubacterium rectale* (*B*), and *Prevotella copri* (*C*). Results for additional species are reported in Supplemental Figures S12–S16, S18–S24.

For *Faecalibacterium prausnitzii* (Sokol et al. 2008; Miquel et al. 2015), 802 distinct strains were detectable in the analyzed samples (Fig. 4A), with only six subjects harboring a strain relatively close (3% SNV rate) to one of the three current isolate genomes. Its genetics were continuously variable and correlated with geography (Fig. 4A); intriguingly, a well-defined subtree of the phylogeny is uniquely composed of strains from the only two non-Westernized populations in this meta-analysis (Peru and Tanzania). *P. copri* showed, conversely, a more discrete population structure, but the resulting SCs were likewise geographically distinct (Fig. 4C). Few strains of *F. prausnitzii* were detected in multiple subjects (13 cases with <1% SNV rate), calling out the degree to which this immune-relevant species is undercharacterized by current isolate sequencing, which has likewise been confirmed by the few isolates’ microbial physiology studies available for this species (Lopez-Siles et al. 2012).

Like *P. copri*, *Eubacterium rectale* strains occurred in distinct SCs forming three genetically distinct groups (Fig. 4B,C). *E. rectale*’s discrete population structure was also confirmed by analysis of the strains’ gene repertoires (Scholz et al. 2016), further strengthening the finding that this species has three distinct subspecies. Interestingly, one of these was specific to the Chinese population, with 71 of its 74 strains derived from the two Chinese sample sets (Qin et al. 2012, 2014). These two studies were independent and carried out using different protocols and commercial kits for sample collection and DNA extraction; therefore, this shows how strain-level analysis is not sensitive to the biases in the same way as quantitative analyses. Likewise, few Chinese samples carried *E. rectale* strains from the other two SCs (1 of the 82 strains in one cluster, and 20 of the 383 strains in the second cluster).

Other strong geographical associations included the three main SCs of *Bacteroides coprocola* (Supplemental Fig. S12) with Spain (72% prevalence in a 68-strain cluster) and China (80% prevalence in a 49-strain cluster), and the structure of *Ruminococcus bromii* (Supplemental Fig. S13). The striking biogeographical patterns of *Eubacterium* species (Supplemental Figs. S14–S16), and especially of *Eubacterium eligens* (a large Chinese subspecies), *Eubacterium hallii* (Spain), and *Eubacterium siraeum* (Denmark and the United States), also suggest that this genus may be particularly prone to population-specific selective pressures. SCs were detected for all prevalent microbial species, and within SCs, strains have very limited genetic diversity (well below 0.1% SNV rate with only very few exceptions) (Supplemental Fig. S17); as expected, inter-SC sequence divergence was instead at least on order of magnitude larger (Supplemental Fig. S17). Like population-specific human genetic alleles, it appears crucial to consider these microbial population structures in future studies of the gut microbiome and its association with host conditions.

### Sets of related strains associate with geography even in otherwise cosmopolitan species

**Figure 5.**
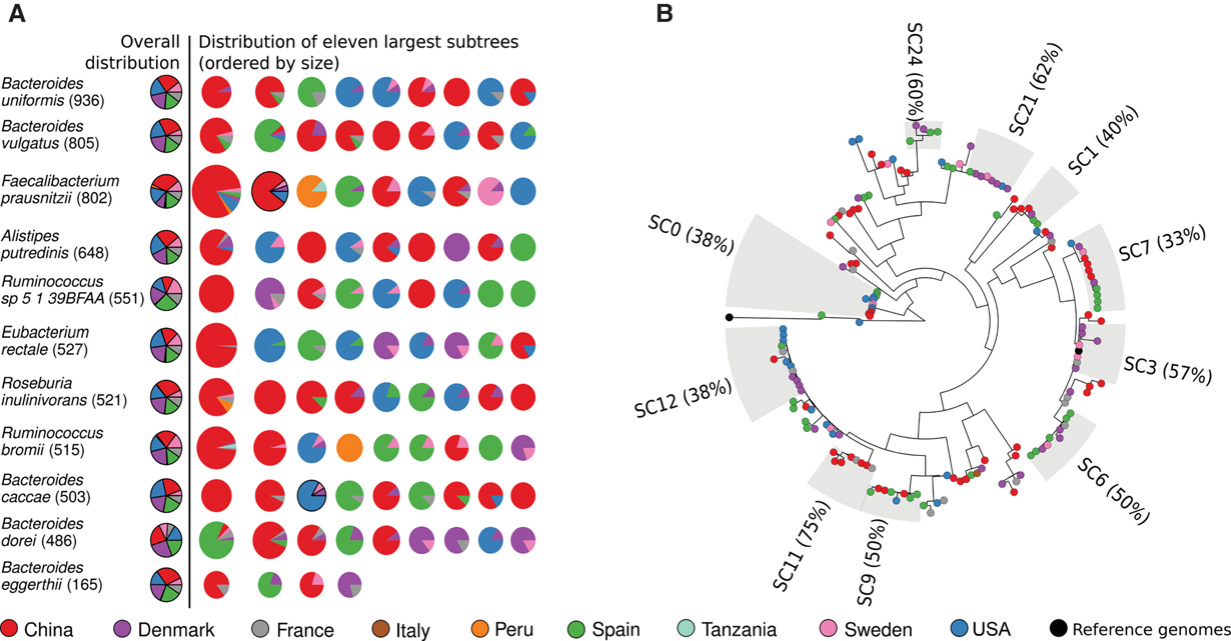
Associations between subspecies clades and geographical location in the 10 most prevalent gut species and *Bacteroides eggerthii*. (*A*) For each of the 10 most prevalent species and *Bacteroides eggerthii* in this sample set, we show the prevalence of each country in the 11 largest subtrees, ordered by size. Subtrees containing reference isolate genomes are marked with a black border. Information regarding subtrees for all species is available as Supplemental Figures S42–S44. (*B*) Example phylogenetic tree of *Bacteroides eggerthii* with the identified subclades.

Even in species lacking strong, geographically discrete SCs, groups of related strains often evidenced significant geographic assortment. The 10 most prevalent species were present in a comparable fraction of subjects in all cohorts and countries, but single phylogenetic subtrees (of at least five strains) were frequently geographically specific (Fig. 5A). *Bacteroides uniformis* (59% overall prevalence) evidenced China-, Spain- and US-specific subtrees among the 11 largest groups (Fig. 5A). Other species have subtrees completely associated with subjects from Denmark (e.g., *Alistipes putredinis*, and partially *E. rectale* and *Bacteroides dorei*), Spain (all the 10 most prevalent species), Peru (*F. prausnitzii* and *Ruminococcus bromii*), France (*Bacteroides vulgatus*), and again China and the United States, for which the number and size of SCs is influenced by the higher number of subjects available for such nations. These country-specific SCs might reflect selection by host genetics or population history, but the tight coclustering of strains of *Butyrivibrio crossotus* (Supplemental Fig. S20) and *F. prausnitzii* (Fig. 4A) in the only two cohorts of non-Westernized population from Peru (Obregon-Tito et al. 2015) and Tanzania (Rampelli et al. 2015) suggests a potentially dominant role of environmental factors such as diet.

Other SCs instead comprised groups of strains with very little genetic diversity (<0.1% of the total species diversity) (Methods) carried by subjects from different continents. For example, SC66 of *Bacteroides caccae* (Supplemental Fig. S25) includes 59 strains with a median of 0.0169% intra-SC SNV rate from the American (24 subjects), Spanish (seven subjects), Chinese (three subjects), Danish (three subjects), and French (four subjects) populations. Their intra-SC SNV rate is much smaller than the minimum (0.045%) and median (0.344%) diversity of SC66 strains compared to other strains in *B. caccae*. Other SCs within this species were likewise shared across populations (e.g., SC61 or SC71), but *B. caccae* also included country-specific clades such as SC41 (12 Chinese strains), SC60 (six Spanish strains), and SC35 (five Danish strains). *Bacteroides eggerthii* also showed similarly genetically related SCs that were geographically diverse (Fig. 5B). The genetic consistency of *B. eggerthii* SCs is striking: For the three largest SCs (SC0, SC6, SC7), the intra-SC median genetic diversities (0.026%, 0.014%, and 0.012%, respectively) were much smaller than the minimum (0.37%, 0.067%, 0.16%) and median genetic distances (0.50%, 0.46%, 0.46%) between the SCs and the other strains. The set of broadly distributed SCs (for additional examples, see Supplemental Figs. S26–S41) thus likely represents key intestinal subspecies that may be important to further characterize by targeted experiments and isolation.

### Genetic diversity of strains in the same species varies significantly for different microbes

It is difficult to define microbial species systematically and to capture each species’ diversity appropriately with reference isolates (Achtman and Wagner 2008; Cordero and Polz 2014); for example, *Streptococcus pneumoniae* universal markers differ by up to 5.0% nucleotide identity across 49 strains, compared to only 1.2% among 15 *Streptococcus mitis* strains (Fraser et al. 2009). StrainPhlAn and the large metagenomic data set we analyzed allowed the assessment of all 125 microbial species’ genetic diversities simultaneously as they occurred in a broad population of human guts, regardless of whether an extensive set of reference genomes was available.

**Figure 6.**
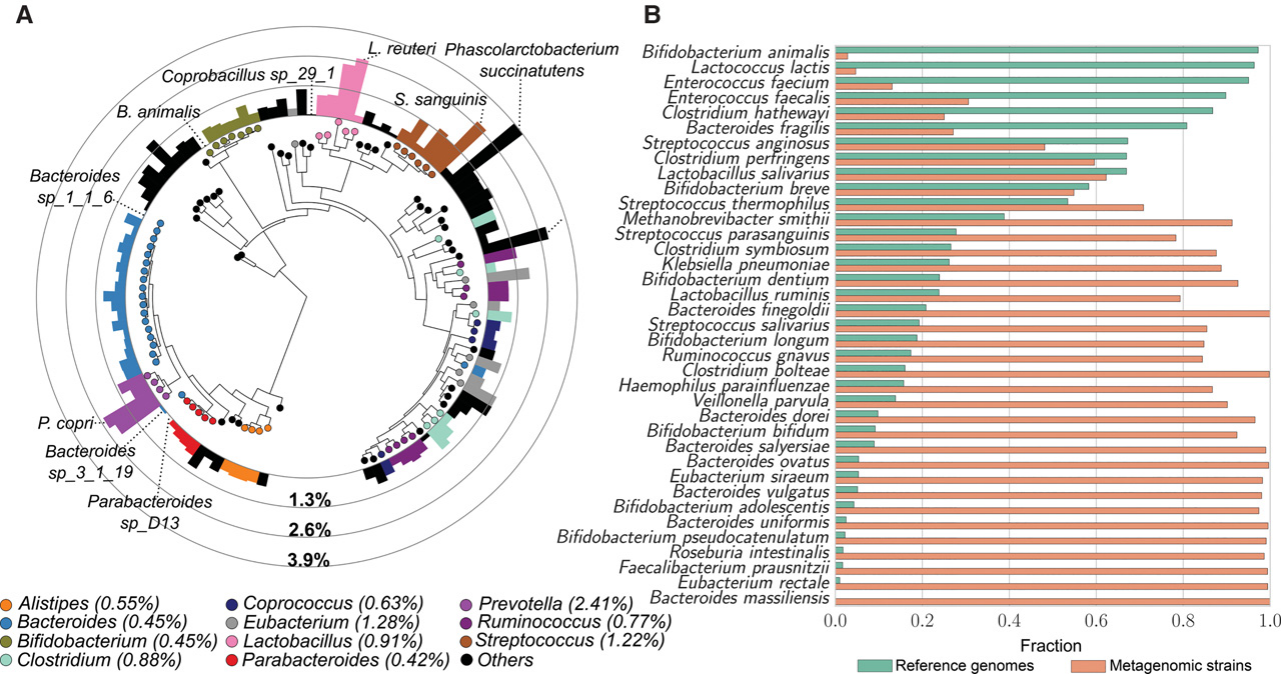
Overall species diversity evaluated across intestinal samples and compared with the diversity available from reference genomes. (*A*) For the 112 species with concatenated marker length >10,000 nt, we built a phylogenetic tree using PhyloPhlAn (Segata et al. 2013) and GraPhlAn (Asnicar et al. 2015) and here report their median SNV rate computed on all pairwise comparisons in this sample set. The median SNV of each genus is reported in parenthesis in the legend. Species diversity ranges between 0.018% (*B. animalis*) and 3.9% (*Phascolarctobacterium succinatutens*) and is partially correlated with phylogeny (*Bacteroides, Parabacteroides, Bifidobacterium*, and *Alistipes* species show consistently lower diversity than *Prevotella*, *Lactobacillus*, and *Streptococcus* species). No significant correlation between diversity and total prevalence or average abundance was observed (Supplemental Fig. S45). Detailed information for each species is reported in Supplemental Table S9. (*B*) Fraction of total branch length spanned by strains sequenced as isolate reference genomes versus branch length spanned by strains from metagenomes. This figure includes species with at least 10 samples, three reference genomes, and concatenated marker length >10,000 nt. The complete set of species is provided in Supplemental Figure S46.

For each species containing at least four strains, we calculated pairwise genetic distances between strains in the same species. The least variable organism was *B. animalis* (0.018% SNV rate), with markers closely matching those of the commercially available probiotic strain. Given this organism's low prevalence and its identity with the sequence of the commercial strain, it is likely that its presence in the human gut typically results from recent probiotic consumption. The most common intestinal genus, *Bacteroides*, comprises species that are generally genetically consistent (Fig. 6A), with diversity indexes as low as 0.36%, 0.37%, and 0.38% for *B. caccae*, *B. intestinalis*, and *B. massiliensis*, respectively. Other *Bacteroides* species are slightly more diverse (*B. coprocola* 0.73%, *B. coprophilus* 0.75%, *B. stercoris* 0.96%) but are still less genetically variable than other prevalent gut microbes, including *Prevotella* species (*P. copri* 2.44%), *F. prausnitzii* (2.94%), *Lactobacillus* (*L. reuteri* 2.74%), *Eubacterium* (*E. siraeum* 1.85%), and some *Ruminococci* (*R. bromii* 1.41%). *Bifidobacteria*, *Parabacteroides*, and *Alistipes* all showed genetic variability in line with that of *Bacteroides*, and all their species have genetic diversities consistent within the corresponding genus (for a full set of diversity indexes, see Supplemental Table S8).

This analysis also revealed many prevalent and/or abundant human gut microbes for which there is a paucity of reference (draft) genomes. Particularly for anaerobic species, we observed genetic diversities between strains more than 10-fold larger than what was previously available (Fig. 6B). These included *Faecalibacterium*, *Roseburia intestinalis*, *E. rectale*, *E. siraeum*, several *Bacteroides* (*B. massiliensis*, *B. ovatus*, *B. salyersiae*, *B. uniformis*, *B. vulgatus*) and *Bifidobacteria* (*B. adolescentis*, *B. bifidum*, *B. pseudocatenulatum*), and they constituted, on average, 63% (SD 18%) of the gut microbiome. In contrast, some species that are more conveniently cultured showed a higher diversity than what we sampled from the gut (e.g., *Enterococcus faecium*, *Enterococcus faecalis, Bacteroides fragilis*, and some *Clostridia*). This may occur because pathogenic strains (which are arguably more likely to be isolated) are unlikely to be found in healthy conditions or in diseases not associated with single pathogens. Other species with reduced diversity in the gut microbiome included those that are also used for commercial fermentation (bifidobacteria, lactobacilli, *Lactococcus lactis*, *B. fragilis*) and organisms that are more typically found in other environments (e.g., *Lactobacillus saliviarius* and *Streptococcus thermophilus* that are characteristic of the oral microbiome) or nonadult guts (e.g., *Bifidobacterium breve* and *Bifidobacterium dentium* enriched in infants). Overall, the genetic diversity we uncovered here for many common colonizers of the human gut suggests that strain specificity is a crucial component of host and microbial phenotype that has, using previous methods, been difficult to analyze directly.

## Discussion

Here, we have developed a new computational method that enables strain-resolved microbial studies directly from metagenomes and applied it to characterize the population structure of the human gut microbiome across the globe by combining cohorts totaling more than 1500 samples. This approach enables strain-level comparative genetics even for microbes not easily amenable to cultivation, including those constituting a large portion of the typical human microbiome. The method exploits the concept of species-specific marker genes (Segata et al. 2012) that are used as genetic proxies of species to efficiently profile strains within species from metagenomes. By comparing the consensus sequences of such markers across samples, StrainPhlAn reconstructs both phylogenetic and ecological relationships between strains populating distinct microbial communities. The method proved superior to other available methods and can accurately reconstruct strain-level phylogenies as evaluated on a number of semisynthetic and real spike-in samples, although assembly or pangenome-based methods (Sharon et al. 2013; Alneberg et al. 2014; Raveh-Sadka et al. 2015; Scholz et al. 2016) are still required to identify strain-specific gene repertoires. In this study specifically, we phylogenetically profiled thousands of strains from 125 undercharacterized intestinal species.

One of the key biological observations of this study is that only one strain typically dominates each species in the human gut, and retention of this individualized dominant strain over time helps to explain the previously reported stability of the gut microbiome (Schloissnig et al. 2013; Franzosa et al. 2015). Strains from the same species in different subjects were generally genetically distinct and associated with host population structure at multiple levels, with different adaptive histories that shaped different species. Even in microbial species defined taxonomically to span roughly the same degree of phylogenetic divergence, some comprised large, discretely differentiated subspecies clades (e.g., *E. rectale*, *P. copri*), whereas others displayed a genetic continuum with smaller geography-specific subclades (e.g., subclades of *F. prausnitzii* or cosmopolitan strains of *B. eggerthii*). Both of these genetic strategies are in contrast to the tighter genetic control and generally reduced diversity often seen in pathogens, for example, the very low divergence rates of *Mycobacterium tuberculosis* (Ford et al. 2011) or infectious (as opposed to more benign) strains of *Streptococcus pneumoniae* (Kilian et al. 2008) or *Staphylococcus aureus* (Holden et al. 2013).

More broadly, the ability to profile strains directly from metagenomes is a key step toward a systems-level understanding of how members of the human microbiome interact with host physiology. Epidemiology and comparative genomics of pathogen populations from isolates has clearly associated specific strains and geography-specific lineages with enhanced virulence potential (Covacci et al. 1999; Suzuki et al. 2012). It will be similarly crucial to associate the presence of strains or subclades of microbial species with immune or chronic disease phenotypes even in the absence of acute infection. The same types of approaches can also start to unravel how members of the microbiome without overt phenotypes are transmitted among hosts, e.g., in vertical mother-to-infant transmission (Milani et al. 2015; Asnicar et al. 2017) or horizontal orofecal routes (Parsonnet et al. 1999). This is of particular interest in the context of interventions such as probiotics or fecal microbiome transplants, in which strain tracking is necessary to identify successful receipt or engraftment of the intended microbes (Li et al. 2016).

Culture-independent strain identification and tracking will also support increasingly high-throughput analyses in microbial ecology. Our finding that a single strain usually dominates per species in the human gut suggests fine-grained microbial competition that might be modifiable by pharmaceutical, nutritional, or environmental interventions. We have investigated only the human gut environment in this study, making it possible that this is a property specific to that or other host-associated environments, and it would be of interest to test the same hypothesis in other microbial communities. The species-specific genetic structures we characterized also imply multiple evolutionary strategies by which individual microbes adapt and incorporate into communities. Discrete subspecies may result from vertical convergent evolution with low horizontal gene flow, whereas species without distinct subclade boundaries (e.g., *F. prausnitzii*) are likely the results of more plastic genomes subject to recombination and lateral gene exchange. This has been described in a few specific cases such as oral neisseriae (Donati et al. 2016), but the relative ease with which thousands of metagenomes can now be obtained compared to isolate (Browne et al. 2016) or single-cell (Gawad et al. 2016) sequencing makes StrainPhlAn profiling of large metagenomes collections a key tool for the understanding of the ecology of the human gut and other microbial communities.

## Methods

StrainPhlAn infers the strain-level phylogenetic structure of microbial species across metagenomic samples by reconstructing the consensus sequences of the dominant strain for each detected species in a sample and then comparing the consensus sequences in different samples (Supplemental Fig. S1). As input, the method takes metagenomic samples and a species-specific marker set, in this case using the markers calculated for MetaPhlAn2 (Truong et al. 2015). Metagenomic reads are aligned to the marker genes, and a consensus sequence is built for each marker. Then, for each species, the consensus sequences in each sample are aligned and concatenated. The concatenated alignments are then used to produce phylogenetic trees using the maximum-likelihood reconstruction principle. Downstream visualization and ordination plots provided directly in the StrainPhlAn package include ordination and subphylogeny analysis and allow cross referencing the inferred phylogenies with available sample metadata. The user can also choose to include in the phylogenies available reference genomes that are useful for providing context for the strains found in the metagenomic samples.

### The StrainPhlAn algorithm

To execute the overall workflow described above, metagenomic reads in each sample are first mapped against the species-specific MetaPlAn2 markers using Bowtie 2 (Langmead and Salzberg 2012). The resulting alignments are processed with BAMtools (Li et al. 2009) to estimate the consensus sequence of each detected species-specific marker. This is performed using a simple majority rule to infer each nucleotide of the markers. Strain-specific markers can also be extracted from available reference genomes (using BLASTN) (Altschul et al. 1990) to include them in the downstream analysis, if chosen by the user.

A number of post-processing operations are then applied in order to perform multiple sequence alignment on high-quality consensus sequences and concatenate them in consistent larger alignments for each species. Specifically, reconstructed markers with a percentage of ambiguous bases (resulting from low-confidence majority rule application or lack of coverage for some regions of the maker) >20% are discarded. Consensus sequences are then trimmed by removing the first and last *n* bases (parameter “–marker_strip_length”, default 50), because the terminal positions are affected by lower coverages due to the limitations in mapping reads against truncated sequences. Strain profiling in a sample, by default, is only provided for species in which the number of reconstructed markers exceeds 80% of the total number of markers available for that species in the MetaPhlAn2 database (this threshold can be defined by the user with the “–marker_in_clade” parameter). After these steps, the reconstructed markers from each metagenomic sample, and if chosen by the user those from the reference genomes, are aligned using MUSCLE (Edgar 2004).

For each marker, the resulting multiple sequence alignments are then processed to remove poorly covered regions. First, both ends of the alignment are trimmed until the fraction of gaps in each position is <20% (parameter “–-gap_in_trailing_col”, default 20%). Second, regions across the remaining alignment that are present in only a small fraction of samples, <30%, are also removed (parameter “–gap_in_internal_col”, default 30%). Third, if the number of the alignment columns with at least one ambiguous nucleotide (i.e., “Ns”) is <80% of the total number of columns (parameter “–N_col”, default 80%), the columns with ambiguous nucleotides are removed. After these steps, the remaining ambiguous nucleotides (“Ns”) in the alignment are replaced with gaps to meet the requirements of the phylogeny reconstruction software.

Next, the processed multiple sequence alignments, for each of the target species, are concatenated. Comparing the concatenated alignment across samples, if the number of long-gap positions (i.e., at least three continuous gap positions) in the concatenated alignment is <80% of the total length (parameter “–-long_gap_percentage”, default 80%), we remove the corresponding columns. Finally, strains that have gaps in >20% of the alignment (parameter “–gap_in_sample”, default 20%) are also removed from the alignment. The edited concatenated alignment is then processed with the maximum-likelihood phylogenetic inference software RAXML (Ott et al. 2007) to produce the phylogenetic trees. Custom scripts are available in our package to build the ordination plots and the heatmaps of genetic-distance matrices. The metadata information is then added to these plots for supporting the discovery of new associations with the population structure of the species (using the script add_metadata.py).

StrainPhlAn required an average of 20 min on a single CPU for profiling all strains in a single high-depth metagenomic sample (averages computed across all the more than 1590 samples analyzed that comprise, on average, ∼5.8 Gb). This is in addition to the prerequisite MetaPhlAn2 step (111 min per CPU). In our analysis, a total of 10 h (single CPU) was required to reconstruct the strain-level phylogeny (including sequence merging, multiple-sequence alignment, and maximum-likelihood-based phylogenetic inference) for each of the 125 species analyzed across the entire 1590 gut metagenomic data set.

### Polymorphic site identification

To identify and study the presence of multiple strains from the same species in a single sample, we investigated the reads-to-markers mapping and sought evidence of polymorphic sites on the alignments suggestive of multiple alleles. To this end, we defined, for each position *s* on the alignment of the reads against the *N*_*s*_ as the total number of reads covering it and *T*_*s*_ as the number of reads supporting the dominant (i.e., most abundant) allele. Given the sequencing error rate *E*, we reject the nonpolymorphic null hypothesis if the probability that the number *N*_*s*_ − *T*_*s*_ of reads coming from the nondominant allele is <α = 0.05. This is estimated with $P_{X\tilde{B}(N_{s},\;1-E)}(X\leq T_{s})$, where *B*(*N*_*s*_,1 − *E*) is the probability mass function of a binomial distribution with *N*_*s*_ trials and the successful rate 1 − *E*. We set the error rate *E* to 0.01 (i.e., 1%) for Illumina sequencing. Failing to reject the null hypothesis reflects the absence of alternative alleles or inability of distinguishing between low-coverage potential alternative alleles and sequencing noise. To further minimize the impact of noise, we remove the bases with quality below 30 before applying the statistical test. To summarize the polymorphic site probabilities at the species level (thus marking the probabilities of multiple sites and markers), we define a polymorphic species as a species having a polymorphic rate greater than µ_polymorphic_rate_ + σ_polymorphic_rate_ where µ_polymorphic_rate_ and σ_polymorphic_rate_ are the median and standard deviation of the polymorphic site across samples, respectively.

### Retention rate and subclade computation

For each species, we computed the rate of single-nucleotide variants (SNVs) between the dominant strains in different samples. The intra-individual SNV rate was calculated for the HMP and MetaHIT data sets, because they are the only considered data sets with multiple samples from the same subjects. The SNV rates for each species was normalized by the median of the inter-everyone comparisons for that species. The resulting distribution is bimodal and represents the distribution of variations between same strains in different samples (values close to zero) and different strains (values centered in the normalized median, i.e., 1.0). For identifying the bimodal distributions, we fitted a two-component Gaussian mixture model and separated the dominant component in the ranges [−∞, µ + 3σ] or [µ − 3σ, +∞].

Country-specific subtrees for Supplemental Figures S9–S20 are computed as the largest subtrees with at least 80% of samples coming from a single country. For identifying the clusters in the principal coordinate plots (Fig. 4), we used the SpectralClustering algorithm implemented in Scikit-learn (Pedregosa et al. 2011) applied on the first two principal coordinates. Subclades for Figure 5B and Supplemental Figures S21–S37 are the largest subtrees in each phylogeny with the largest intra-SNVs rate <0.1%. In addition, a subclade must have strains from at least two subjects or contain at least one reference genome and one strain in a sample.

### Data collection and preprocessing

In total, 1590 publicly available gut metagenomics samples comprising nine human-associated data sets were considered in this work (Table 1). Of these studies, seven were associated with human disease and two from healthy cohorts. The cohort data sets spanned geographic locations from all continents (except Australia and Antarctica), and two included non-Westernized populations from Peru and Tanzania. All data sets are cross sectional, with the exception of two cohorts (MetaHIT and HMP), which included longitudinal sampling of the same individuals over a period of 163 SD 125 d and 219 SD 69 d, respectively. When the same sequenced samples were originally included in more than one study, i.e., some samples from the Obesity data set (Le Chatelier et al. 2013) are present also in the MetaHIT data set (Nielsen et al. 2014), we considered them only once in our combined data set.

All samples were preprocessed by the standard HMP quality control procedure (Human Microbiome Consortium 2012), and reads shorter than the thresholds reported in Table 1 were removed. Taxonomic profiling to identify which microbial species are present and at what abundance in each sample, was performed with MetaPhlAn2 (Truong et al. 2015).

### Method validation and evaluation

For validation, StrainPhlAn was applied to a combination of synthetic and semisynthetic data sets. StrainPhlAn was first tested on two HMP Mock samples (Human Microbiome Consortium 2012) containing strains from 21 known reference genomes, in which their abundances were either staggered or evenly distributed. StrainPhlAn reconstructed the strains for the 11 species with sufficient coverage (Supplemental Table S1). Except for *Staphylococcus aureus* and *Clostridium beijerinckii* (whose genomes are discordant also based on metagenomic assembly) (Supplemental Table S1), our method can reconstruct the other species strains with SNV rates less than 0.0001.

In addition, we also validated StrainPhlAn on 36 synthetic data sets of four species (*Bacteroides dorei, Bacteroides fragilis, Bacteroides ovatus, Bifidobacterium longum*). For each species, we generated synthetic data by sampling reads from its genomes with an Illumina-based error model (McElroy et al. 2012) with coverages ranging from 2× to 10× using custom scripts available at https://bitbucket.org/CibioCM/synmetap/overview. These synthetic samples were then also added to real HMP stool metagenomes (in which the four synthetic species was absent) to create 36 additional semisynthetic samples. StrainPhlAn was applied on both synthetic and semisynthetic samples, and the accuracy was evaluated by detecting the number of SNVs of the reconstructed markers compared to the original reference genomes. The evaluation was repeated at increasing coverages of the target strains as reported in Supplemental Figure S2. An additional validation was performed by reconstructing strain markers from synthetic metagenomes and including them in the phylogeny built with the reference genomes (Supplemental Figs. S3, S4). On the combined phylogeny, the accuracy of the reconstruction can be evaluated by measuring the phylogenetic distance between the reconstructed strains and the corresponding reference genome (Supplemental Figs. S3, S4). ConStrains (Luo et al. 2015) was applied on the same data (Supplemental Figs. S3, S4). For the validation on real samples (Supplemental Fig. S5; Supplemental Table S2), we used 19 metagenomes in the MetaHIT (Nielsen et al. 2014) data set from subjects that consumed a fermented milk product containing the previously sequenced *Bifidobacterium animalis* subsp. *lactis CNCM I-2494*.

### Software availability

StrainPhlAn (version 1.0) is implemented in Python within the MetaPhlAn2 package (version 2.5.0) and is available with source code, manual, tutorials, and a support user group at http://segatalab.cibio.unitn.it/tools/strainphlan and in the updated MetaPhlAn2 repository at http://segatalab.cibio.unitn.it/tools/metaphlan2/. The code and necessary supporting databases are maintained in a Bitbucket repository at https://bitbucket.org/biobakery/metaphlan2. A snapshot of the implementation used in this work is also available as Supplementary Code, without, however, the large database files that are maintained at https://bitbucket.org/biobakery/metaphlan2.

## Supplementary Material

Supplemental Material
