# Supplementary material for: Microbial strain-level population structure and genetic diversity from metagenomes
Source: Genome Res. 2017 Apr;27(4):626–38. doi: 10.1101/gr.216242.116 (PMC5378180; doi:10.1101/gr.216242.116)
Supplement: Supplemental Material [file supp_27_4_626__index.html]

Microbial strain-level population structure and genetic diversity from metagenomes — Supplemental Material 

# Microbial strain-level population structure and genetic diversity from metagenomes

## Supplemental Material

- Supplemental\_Table\_S8.xlsx
- Supplemental\_Table\_S9.xlsx
- Supplementary\_Code.tar.gz
- Supplemental\_Material.pdf
