# Supplementary material for: Microbial strain-level population structure and genetic diversity from metagenomes
Source: Genome Res. 2017 Apr;27(4):626–38. doi: 10.1101/gr.216242.116 (PMC5378180; doi:10.1101/gr.216242.116)
Supplement: Supplemental Material [file supp_gr.216242.116_Supplemental_Material.pdf]

Microbial strain-level population structure and genetic diversity from metagenomes

Duy Tin Truong[1], Adrian Tett[1], Edoardo Pasolli[1], Curtis Huttenhower[2,3], Nicola Segata[1]

1 Centre for Integrative Biology, University of Trento, Trento, Italy
2 Biostatistics Department, Harvard School of Public Health, Boston, MA, USA
3 The Broad Institute, Cambridge, MA, USA

Corresponding author: Nicola Segata (nicola.segata@unitn.it)

Supporting Information

Contents

1. Supplemental Tables

Supplemental Table S1. Comparison of the markers reconstructed from the HMP mock communities with StrainPhlAn and MIDAS, versus the markers from the known reference genomes used to build the mock communities 5

Supplemental Table S2. The comparison of StrainPhlAn and MIDAS performance on the synthetic dataset of *Bacteroides dorei* 20x coverage 5

Supplemental Table S3. The comparison of StrainPhlAn and MIDAS performance on the synthetic dataset of *Bacteroides fragilis* 20x coverage 6

Supplemental Table S4. The comparison of StrainPhlAn and MIDAS performance on the synthetic dataset of *Bacteroides ovatus* 20x coverage 6

Supplemental Table S5. The comparison of StrainPhlAn and MIDAS performance on the synthetic dataset of *Bifidobacterium longum* 20x coverage 6

Supplemental Table S6. SNV rate comparison of the strains reconstructed by StrainPhlAn and MIDAS on 7 MetaHIT samples 7

Supplemental Table S7. StrainPhlAn accurately reconstructs the marker sequences of strains from gut metagenomes 7

Supplemental Table S8. Genetic diversity of strains in the 125 species analyzed. (See Supplemental_Table_S8.xlsx Excel file) ....

Supplemental Table S9. Relative abundance of dominant strains for the 125 species analyzed. (See Supplemental_Table_S9.xlsx Excel file) ....

2. Supplemental Figures

Supplemental Fig. S1. The StrainPhlAn pipeline 8

Supplemental Fig. S2. Marker genes have a genetic variability consistent with that of core genes 9

Supplemental Fig. S3. The distribution of SNV rates of marker genes is consistent with that of core genes 10

Supplemental Fig. S4. StrainPhlAn performance in reconstructing strains from 4 species (*Bacteroides dorei, Bacteroides fragilis, Bacteroides ovatus, Bifidobacterium longum*) using 72 synthetic and semi-synthetic datasets........ 11

Supplemental Fig. S5. The comparison of the strains reconstructed by StrainPhlAn and ConStrains on the synthetic datasets of two species *Bacteroides dorei*, and *Bacteroides fragilis* at 20x coverage 12

Supplemental Fig. S6. The comparison of the strains reconstructed by StrainPhlAn and ConStrains on the synthetic datasets of two species Bacteroides ovatus, and Bifidobacterium longum at 20x coverage 13

Supplemental Fig. S7. Comparison of the *B. animalis* strains reconstructed by StrainPhlAn from real sample with the reference genomes 14

Supplemental Fig. S8. StrainPhlAn reconstructions of B. animalis strains from metagenomes is consistent with the corresponding sequenced genomes 14

Supplemental Fig. S9. Distribution of non-polymorphic site prevalence in samples for the 40 most prevalent gut bacterial species 15

Supplemental Fig. S10. Rates of non-polymorphic positions and within-species dominant strain dominance are not correlated with relative abundance of the species 16

Supplemental Fig. S11. Strain retention rates and strain divergence in multiple longitudinal samples from the same subjects (131 from the HMP and 78 from MetaHIT)........ 17

Supplemental Fig. S12. Population genomics structure of *Bacteroides coprocola* and their associated sampling countries 18

Supplemental Fig. S13. Population genomics structure of *Ruminococcus bromii* and their associated sampling countries 18

Supplemental Fig. S14. Population genomics structure of *Eubacterium eligens* and their associated sampling countries 19

Supplemental Fig. S15. Population genomics structure of *Eubacterium hallii* and their associated sampling countries 19

Supplemental Fig. S16. Population genomics structure of *Eubacterium siraeum* and their associated sampling countries 20

Supplemental Fig. S17. Genetic distances between strains in the same sub-clades (Intra-SCs) and between strains in different sub-clades (Inter-SC) for each of the forty most prevalent gut microbial species........ 21

Supplemental Fig. S18. Population genomics structure of *Bacteroides intestinalis* and their associated sampling countries . 22

Supplemental Fig. S19. Population genomics structure of *Bacteroides massiliensis* and their associated sampling countries 22

Supplemental Fig. S20. Population genomics structure of *Butyrivibrio crossotus* and their associated sampling countries 23

Supplemental Fig. S21. Population genomics structure of *Dialister invisus* and their associated sampling countries 23

Supplemental Fig. S22. Population genomics structure of *Dorea formicigenerans* and their associated sampling countries ... 24

Supplemental Fig. S23. Population genomics structure of *Prevotella stercorea* and their associated sampling countries 24

Supplemental Fig. S24. Population genomics structure of *Ruminococcus lactaris* and their associated sampling countries ... 25

Supplemental Fig. S25. The phylogenetic tree of *Bacteroides caccae* with the identified subspecies-subclades (SC) and their associated sampling countries 25

Supplemental Fig. S26. The phylogentic tree of *Akkermansia muciniphila* with the identified subspecies-subclades (SC) and their associated sampling countries 26

Supplemental Fig. S27. The phylogenetic tree of *Bacteroides cellulosilyticus* with the identified subspecies-subclades (SC) and their associated sampling countries 26

Supplemental Fig. S28. The phylogenetic tree of *Bacteroides coprocola* with the identified subspecies-subclades (SC) and their associated sampling countries 27

Supplemental Fig. S29. The phylogenetic tree of *Bacteroides dorei* with the identified subspecies-subclades (SC) and their associated sampling countries 27

Supplemental Fig. S30. The phylogenetic tree of *Bacteroides eggerthii* with the identified subspecies-subclades (SC) and their associated sampling countries 28

Supplemental Fig. S31. The phylogenetic tree of *Bacteroides faecis* with the identified subspecies-subclades (SC) and their associated sampling countries 28

Supplemental Fig. S32. The phylogenetic tree of *Bacteroides finegoldii* with the identified subspecies-subclades (SC) and their associated sampling countries 29

Supplemental Fig. S33. The phylogenetic tree of Bacteroides massiliensis with the identified subspecies-subclades (SC) and their associated sampling countries 29

Supplemental Fig. S34. The phylogenetic tree of Bacteroides ovatus with the identified subspecies-subclades (SC) and their associated sampling countries 30

Supplemental Fig. S35. The phylogenetic tree of Bacteroides salyersiae with the identified subspecies-subclades (SC) and their associated sampling countries 30

Supplemental Fig. S36. The phylogenetic tree of Bacteroides stercoris with the identified subspecies-subclades (SC) and their associated sampling countries 31

Supplemental Fig. S37. The phylogenetic tree of *Lachnospiraceae bacterium 1_1_57FAA* with the identified subspecies-subclades (SC) and their associated sampling countries 31

Supplemental Fig. S38. The phylogenetic tree of *Parabacteroides distasonis* with the identified subspecies-subclades (SC) and their associated sampling countries 32

Supplemental Fig. S39. The phylogenetic tree of *Parabacteroides johnsonii* with the identified subspecies-subclades (SC) and their associated sampling countries 32

Supplemental Fig. S40. The phylogenetic tree of *Streptococcus thermophiles* with the identified subspecies-subclades (SC) and their associated sampling countries 33

Supplemental Fig. S41. The phylogenetic tree of *Sutterella wadsworthensis* with the identified subspecies-subclades (SC) and their associated sampling countries 33

Supplemental Fig. S42. Subspecies-clades identified for the most prevalent species within the whole sample set of 1,590 metagenomes and their geographical association........ 34

Supplemental Fig. S43. Subspecies-clades identified for the most prevalent species within the whole sample set of 1,590 metagenomes and their geographical association 35

Supplemental Fig. S44. Subspecies-clades identified for the most prevalent species within the whole sample set of 1,590 metagenomes and their geographical association 36

Supplemental Fig. S45. The genetic diversity of different species 37

Supplemental Fig. S46. Fraction of total branch length spanned by strains sequenced in isolation (reference genomes) versus total branch length spanned by strains retrieved from metagenomes of species with at least three reference genomes 38

SUPPLEMENTARY TABLES

Supplemental Table S1. Comparison of the markers reconstructed from the HMP mock communities with StrainPhlAn and MIDAS, versus the markers from the known reference genomes used to build the mock communities. For two mock communities with evenly and staggered distributed abundances, we report the rate of single-nucleotide errors, the absolute number of single nucleotide errors, and the length of the concatenated marker alignment obtained by StrainPhlAn and MIDAS. StrainPhlAn obtained errors below 0.1% for all reconstructed strains except for *Staphylococcus aureus* and *Clostridium beijerinckii*; however, when we performed metagenomic assembly, we confirmed that these two organisms have divergent genomes compared to the reference (only 98.98% and 99.57% average identity for the reconstructed contigs). These two genomes thus represent outliers for biological rather than validation reasons. MIDAS could only reconstruct 3 strains from both mock communities (missing reconstructions are marked with "NA") and it showed substantially higher error rates compared to those of StrainPhlAn. For StrainPhlAn, we compared the sequence of the reconstructed markers against the sequence of the markers of the genomes of the strains used in the mock community (the "true" genomes). For MIDAS we similarly compared the "true" genomes against the reference genome selected by the method edited with the suggested nucleotide variations, limiting the comparison to the regions of the genome that MIDAS reports in the output.

Sample	Target strain	StrainPhlAn			MIDAS		
		Single nucleotide errors		Alignment Length	Single nucleotide errors		Alignment Length
		Rate	Number		Rate	Number	
Evenly distributed mock community (SRR172902)	*Bacteroides vulgatus NC 009614*	0.000079	10	126015	NA	NA	NA
	Clostridium beijerinckii NC 009617	0.001138	112	98399	NA	NA	NA
	Staphylococcus aureus NC 010079	0.008175	593	72534	NA	NA	NA
	Deinococcus radiodurans NC 001263	0.000062	5	81150	0.01006	31717	3152738
	Acinetobacter baumannii NC 009085	0.000196	12	61321	0.038153	113326	2970342
	Rhodobacter sphaeroides NC 007493	0.000991	39	39340	NA	NA	NA
	Staphylococcus epidermidis NC 004461	0.000131	11	84135	0.017169	31095	1811080
	Propionibacterium acnes NC 006085	0.000223	22	98714	NA	NA	NA
	Streptococcus mutans NC 004350	0.000730	57	78092	NA	NA	NA
	Actinomyces odontolyticus NZ DS264586	0.000483	39	80715	NA	NA	NA
Staggered distributed mock community (SRR172903)	*Staphylococcus aureus NC 010079*	0.008738	729	83430	0.047408	120755	2547166
	Rhodobacter sphaeroides NC 007493	0.000056	4	72010	NA	NA	NA
	Staphylococcus epidermidis NC 004461	0.000034	3	88239	0.026905	61252	2276621
	Streptococcus mutans NC 004350	0.000011	1	89221	0.008452	15702	1857847
	Escherichia coli NC 000913	0.000185	2	10796	NA	NA	NA

Supplemental Table S2. The comparison of StrainPhlAn and MIDAS performance on the synthetic dataset of *Bacteroides dorei* 20x coverage. We report the alignment length of strains obtained by two methods and the single nucleotide variant (SNV) between them and the reference genomes. MIDAS failed to reconstruct all *Bacteroides dorei* strains.

	**StrainPhlAn**	**MIDAS**
Alignment length	83393	NA
**Genome**	**StrainPhlAn SNV rate**	**MIDAS SNV rate**
G000156075	3.60E-05	NA
G000158335	0	NA
G000273035	0	NA
G000273055	0	NA
G000273075	0	NA
G000738045	0	NA
G000738065	0	NA

Supplemental Table S3. The comparison of StrainPhlAn and MIDAS performance on the synthetic dataset of *Bacteroides fragilis* 20x coverage. We report the alignment length of strains obtained by two methods and the single nucleotide variant (SNV) between them and the reference genomes. StrainPhlAn outperforms MIDAS with much smaller SNVs on all genomes.

	**StrainPhlAn**	**MIDAS**
Alignment length	76304	3426398
**Genome**	**StrainPhlAn SNVs rate**	**MIDAS SNVs rate**
G000273155	3.90E-05	0.026449
G000598145	9.20E-05	0.018223
G000598225	3.90E-05	0.008044
G000598385	6.60E-05	0.011365
G000598425	3.90E-05	0.028594
G000598505	0.000118	0.027205
G000598665	0	0.015147
G000598805	5.20E-05	0.017314
G000599305	3.90E-05	0.010108
G000601055	6.60E-05	0.005212

Supplemental Table S4. The comparison of StrainPhlAn and MIDAS performance on the synthetic dataset of *Bacteroides ovatus* 20x coverage. We report the alignment length of strains obtained by two methods and the single nucleotide variant (SNV) between them and the reference genomes. StrainPhlan outperforms MIDAS with much smaller SNVs on all genomes.

	**StrainPhlAn**	**MIDAS**
Alignment length	52113	1951064
**Genome**	**StrainPhlAn SNVs rate**	**MIDAS SNVs rate**
G000154125	0	0.008573
G000178275	0	0.012007
G000218325	0	0.008606
G000273195	0	0.002943
G000273215	0	0.009838
G000699665	0	0.003794
G000699725	0	0.009572

Supplemental Table S5. The comparison of StrainPhlAn and MIDAS performance on the synthetic dataset of *Bifidobacterium longum* 20x coverage. We report the alignment length of strains obtained by two methods and the single nucleotide variant (SNV) between them and the reference genomes. StrainPhlan outperforms MIDAS with much smaller SNVs on all genomes.

	**StrainPhlAn**	**MIDAS**
Alignment length	112404	1693645
**Genome**	**StrainPhlAn SNVs rate**	**MIDAS SNVs rate**
G000261205	0	0.007741
G000261225	0	0.013356
G000261245	0	0.002565
G000261265	0	0.006775
G000478525	1.80E-05	0.01061
G000730025	0	0.015427
G000730035	0	0.001752
G000730055	2.70E-05	0.012826
G000730105	1.80E-05	0.006472
G000730135	0	0.008965

Supplemental Table S6. SNV rate comparison of the strains reconstructed by StrainPhlAn and MIDAS on 7 MetaHIT samples. These 7 samples are from subjects that consumed a predefined fermented milk product containing *Bifidobacterium animalis subsp. lactis CNCM I-2494* whose genome is publicly available. The SNV rates were computed between the reconstructed strains and the genome of *Bifidobacterium animalis subsp. lactis CNCM I-2494*. StrainPhlAn obtained much smaller SNV rates in all cases compared to those of MIDAS.

	StrainPhlAn			MIDAS			
Alignment length	37069			1172344			
Sample ID	O2_UC20_2	O2_UC30_2	O2_UC24_2	O2_UC47_2	O2_UC32_2	O2_UC37_2	O2_UC23_2
StrainPhlAn	0.000108	0.00027	0.000081	0	0.000189	0.00027	0.000081
MIDAS	0.009448	0.014675	0.008573	0.047576	0.015637	0.007871	0.014611

Supplemental Table S7. StrainPhlAn accurately reconstructs the marker sequences of strains from gut metagenomes. We evaluated the accuracy of StrainPhlAn in reconstructing the markers of the genome of *Bifidobacterium animalis subsp. lactis CNCM I-2494* in 7 MetaHIT samples (the subset of the 19 individuals subjected to *B. animalis* intake in which this species is present in the metagenome at >2x coverage). These 7 samples are from subjects that consumed a predefined fermented milk product containing *Bifidobacterium animalis subsp. lactis CNCM I-2494* whose genome is publicly available. Our pipeline reconstructed the targeted strain for the 7 samples with coverage >2x achieving less than 0.01% single nucleotide errors, which is more than ten times smaller than the average nucleotide variation observed between strains (1.3%) computed on the markers from the sequenced reference genomes in this species (Figs. S5 and S6).

Sample ID	O2_UC32_2	O2_UC30_2	O2_UC24_2	O2_UC20_2	O2_UC23_2	O2_UC37_2	O2_UC47_2	*B. animalis I-2494*
O2_UC32_2	0	17	10	11	10	17	7	7
O2_UC30_2	17	0	13	14	13	20	10	10
O2_UC24_2	10	13	0	7	6	13	3	3
O2_UC20_2	11	14	7	0	7	14	4	4
O2_UC23_2	10	13	6	7	0	13	3	3
O2_UC37_2	17	20	13	14	13	0	10	10
O2_UC47_2	7	10	3	4	3	10	0	0
B. animalis I-2494	7	10	3	4	3	10	0	0

Supplemental Table S8. Genetic diversity of strains in the 125 species analyzed. (See Supplemental_Table_S8.xlsx Excel file)

Supplemental Table S9. Relative abundance of dominant strains for the 125 species analyzed. (See Supplemental_Table_S9.xlsx Excel file)

SUPPLEMENTARY FIGURES

Supplemental Fig. S1. The StrainPhlAn pipeline. The input for StrainPhlAn is a set of metagenomic samples and, optionally, a collection of reference genomes. StrainPhlAn then reconstructs all dominant strains present in the samples by mapping the reads against the MetaPhlAn2 markers with Bowtie2 (8) and reconstructing the sample-specific consensus sequence for all the markers. Markers are also extracted form reference genomes (if provided by the user) using Blastn (10). For each species, the pipeline then reconstructs the concatenated multiple sequence alignment from the single consensus sequences and uses it to build the phylogenetic tree. Other output formats include ordination plots and heatmaps representing strain-level genetic relations.

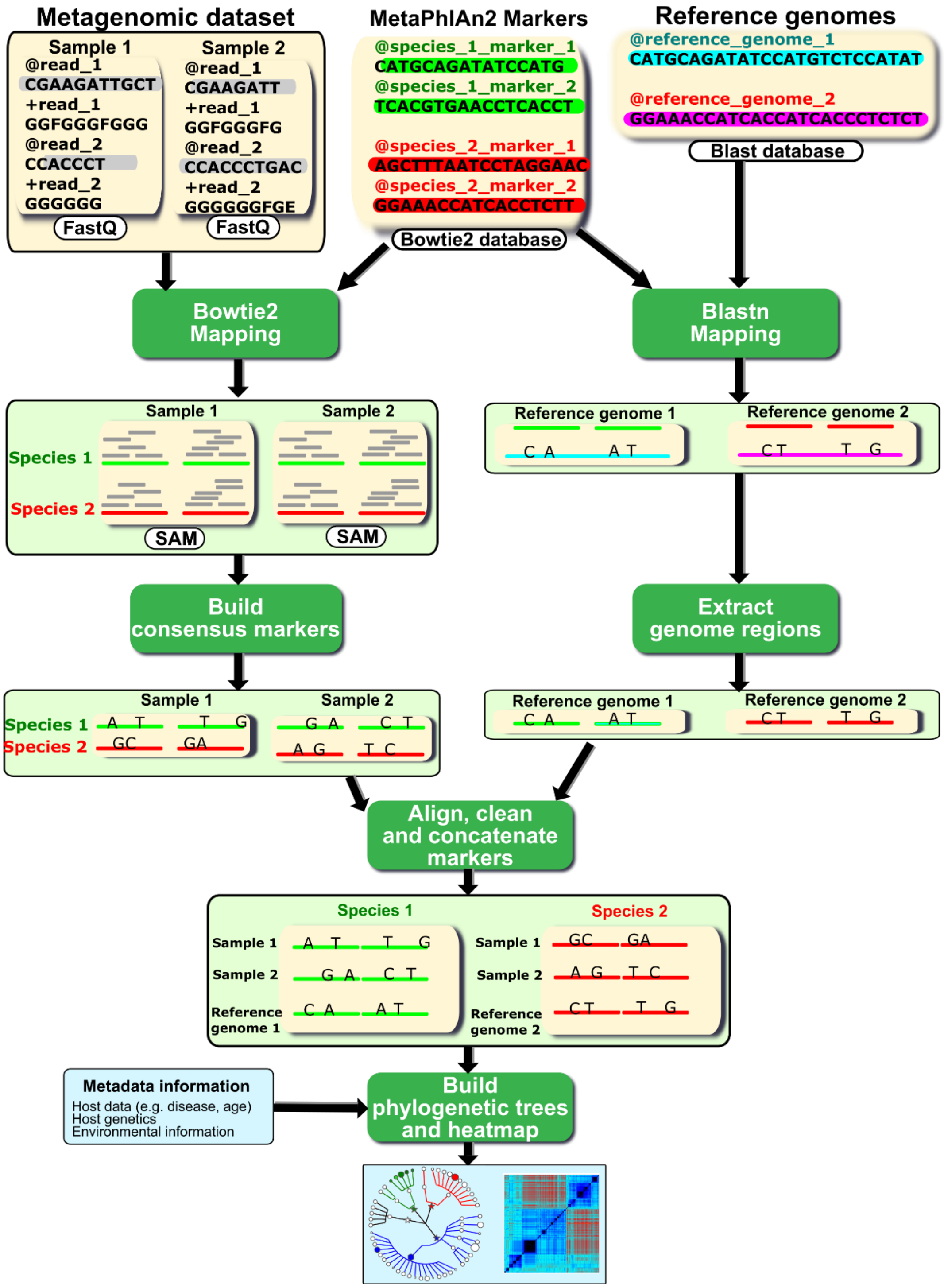

Supplemental Fig. S2. Marker genes have a genetic variability consistent with that of core genes. For the 32 most prevalent gut microbiome species with at least three sequenced genomes, we analyzed the SNV variability of core genes (genes present in all the genomes in a species at >90% percentage identity) and the SNV variability of the marker genes used by StrainPhlAn. The boxplot reports the median SNV rate of each core or marker gene across the pairs of orthologues of the genes in the available reference genomes. In only 7 cases (asterisk appended to the species name) the two-sided Kolmogorov-Smirnov statistical test found that SNV variabilities associated with core genes and marker genes have a significantly difference distribution, but the effect size of the variation is still small.

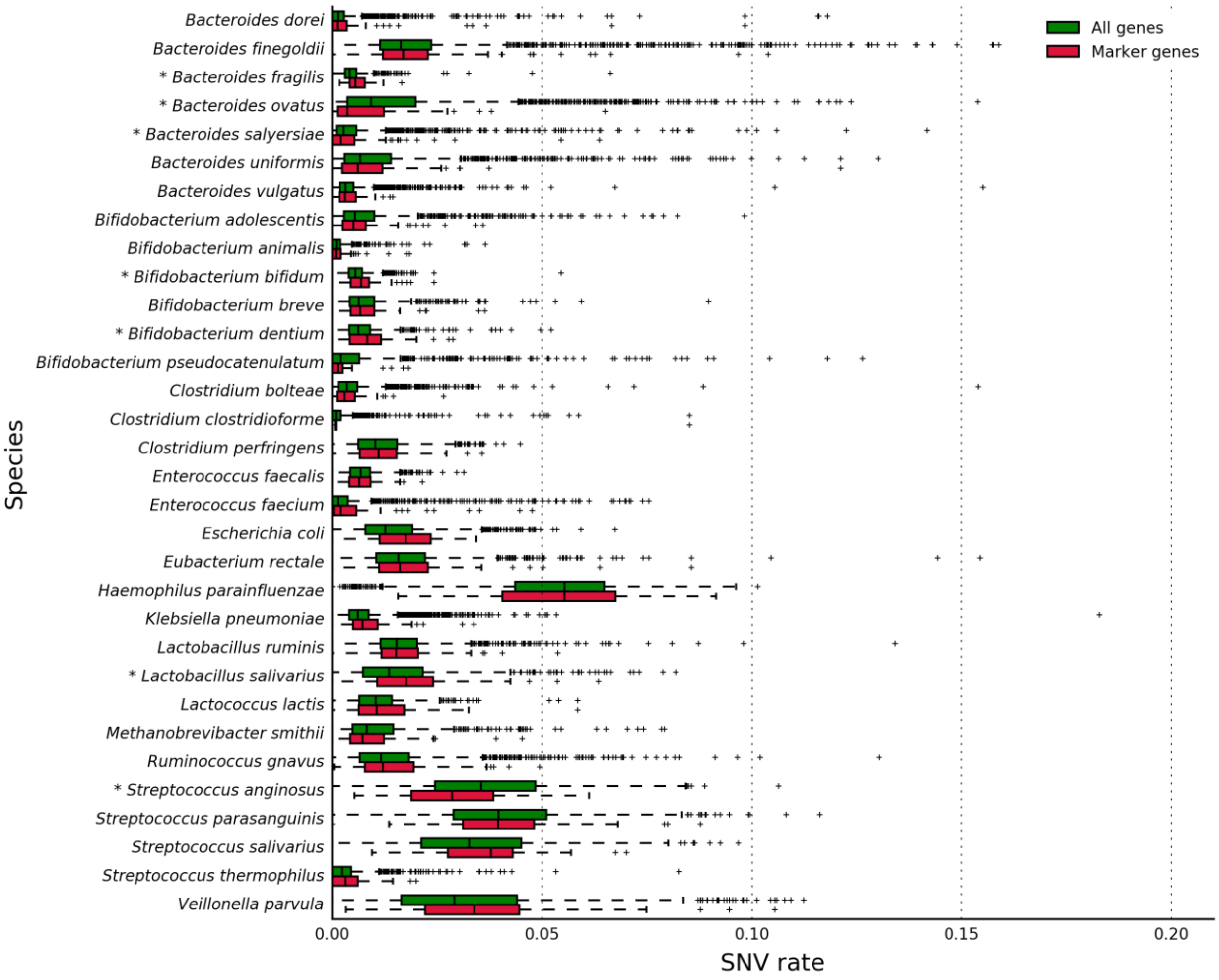

Supplemental Fig. S3. The distribution of SNV rates of marker genes is consistent with that of core genes. We report here the histograms of the genetic variability of marker genes and core genes computed as reported in Supplemental Fig. S2. The histograms confirm that the genetic variability of marker genes and core genes is distributed consistently. It also highlights that, for some species, a considerable fraction of core (and marker) genes show almost no variations in different strains, although this may be the consequence of a reduced diversity of the strains with sequenced genomes.

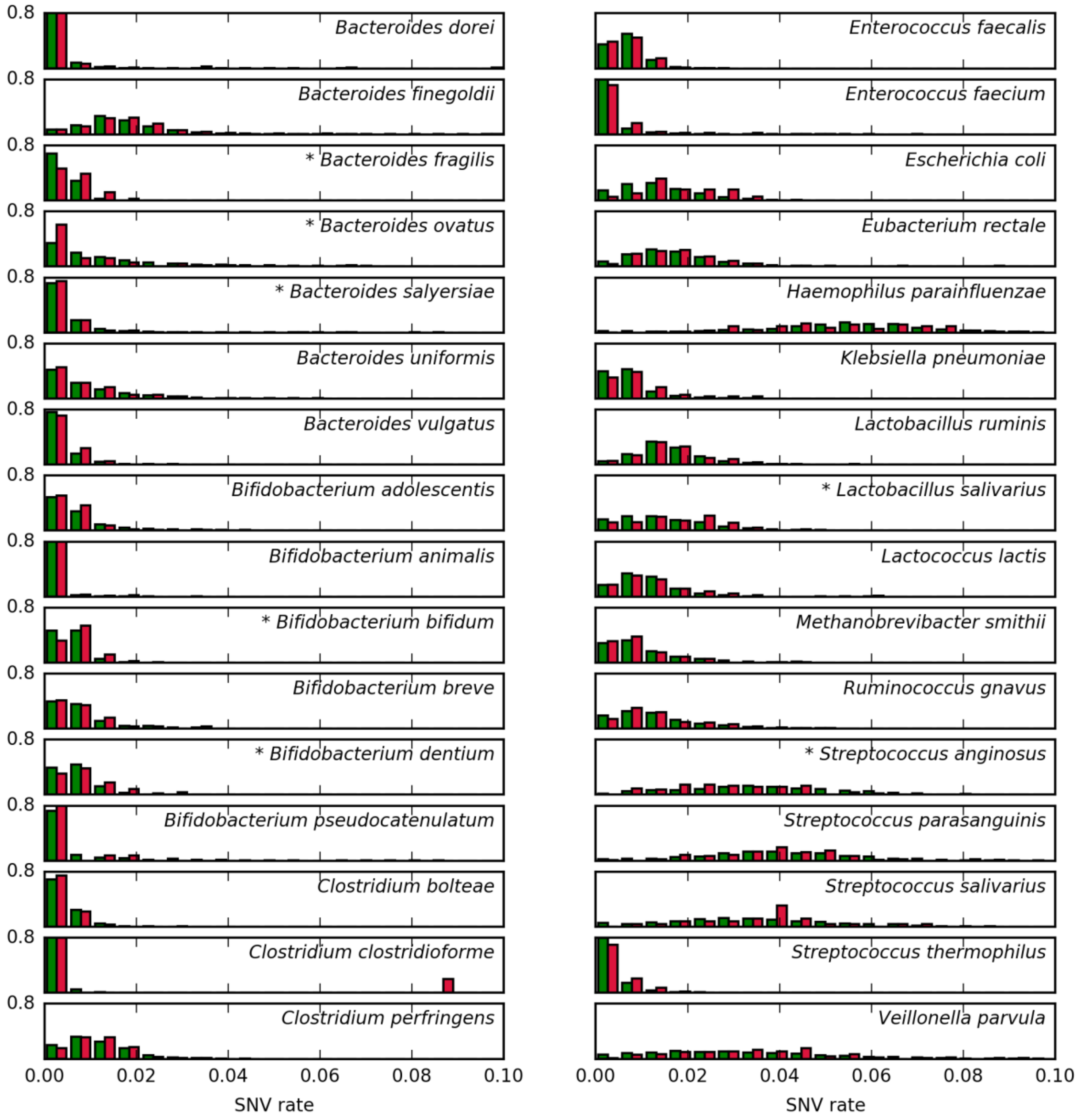

Supplemental Fig. S4. StrainPhlAn performance in reconstructing strains from 4 species (*Bacteroides dorei, Bacteroides fragilis, Bacteroides ovatus, Bifidobacterium longum*) using 72 synthetic and semi-synthetic datasets. The synthetic samples were generated from the reference genomes of the four target species with simulated sequencing noise and confounding non-target species at increasing coverages (**see Methods**). Semi-synthetic datasets also include a large fraction of reads from real gut metagenomes (**see Methods**). The boxplots show the distances (in nucleotide difference rates) between the reconstructed markers of target strains from synthetic and semi-synthetic samples and the reference genomes of the target strains. StrainPhlAn could recover the strains precisely (with the distance of <0.025% SNVs rate) even at coverages as low as 3x for all species. Additionally, the SNV rates tend to converge to zero when increasing the coverage.

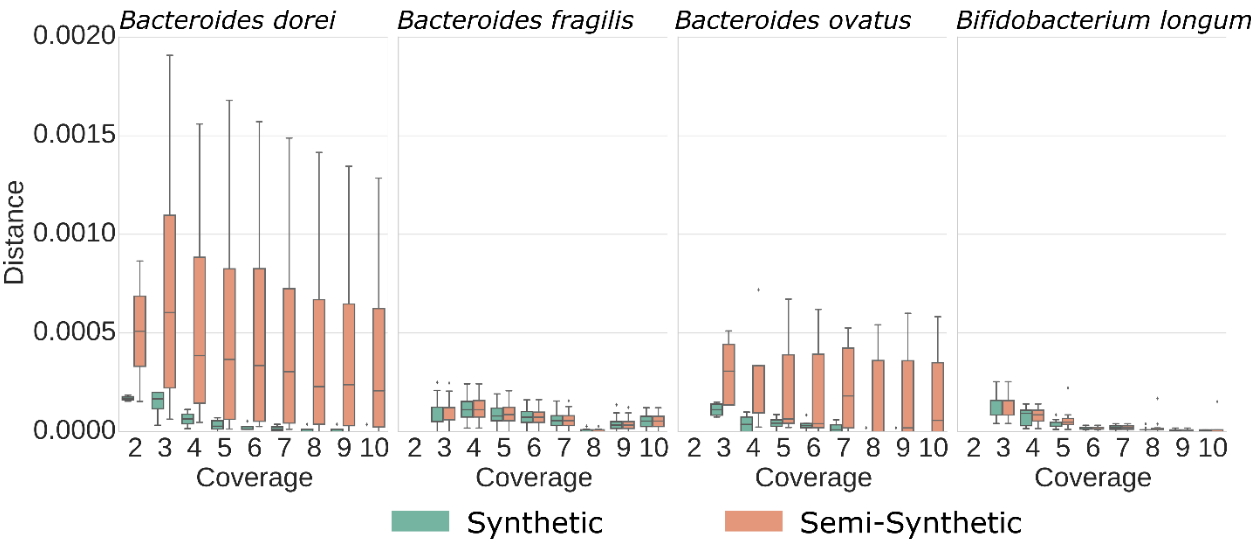

Supplemental Fig. S5. The comparison of the strains reconstructed by StrainPhlAn and ConStrains on the synthetic datasets of two species *Bacteroides dorei*, and *Bacteroides fragilis* at 20x coverage. (A, C) The phylogenetic trees built from reference genomes and from corresponding synthetic samples by StrainPhlAn. In parenthesis, we report the strain IDs assigned by ConStrains. (B, D) To confirm the sequence divergence between the strains in the tree, we report the single nucleotide variant (SNV) distance matrix between the reference genomes used in panels A and C. These distances were computed on the whole set of core genes as obtained by the application of Roary (6). Genomes were labeled also here according to the ConStrains strain prediction.

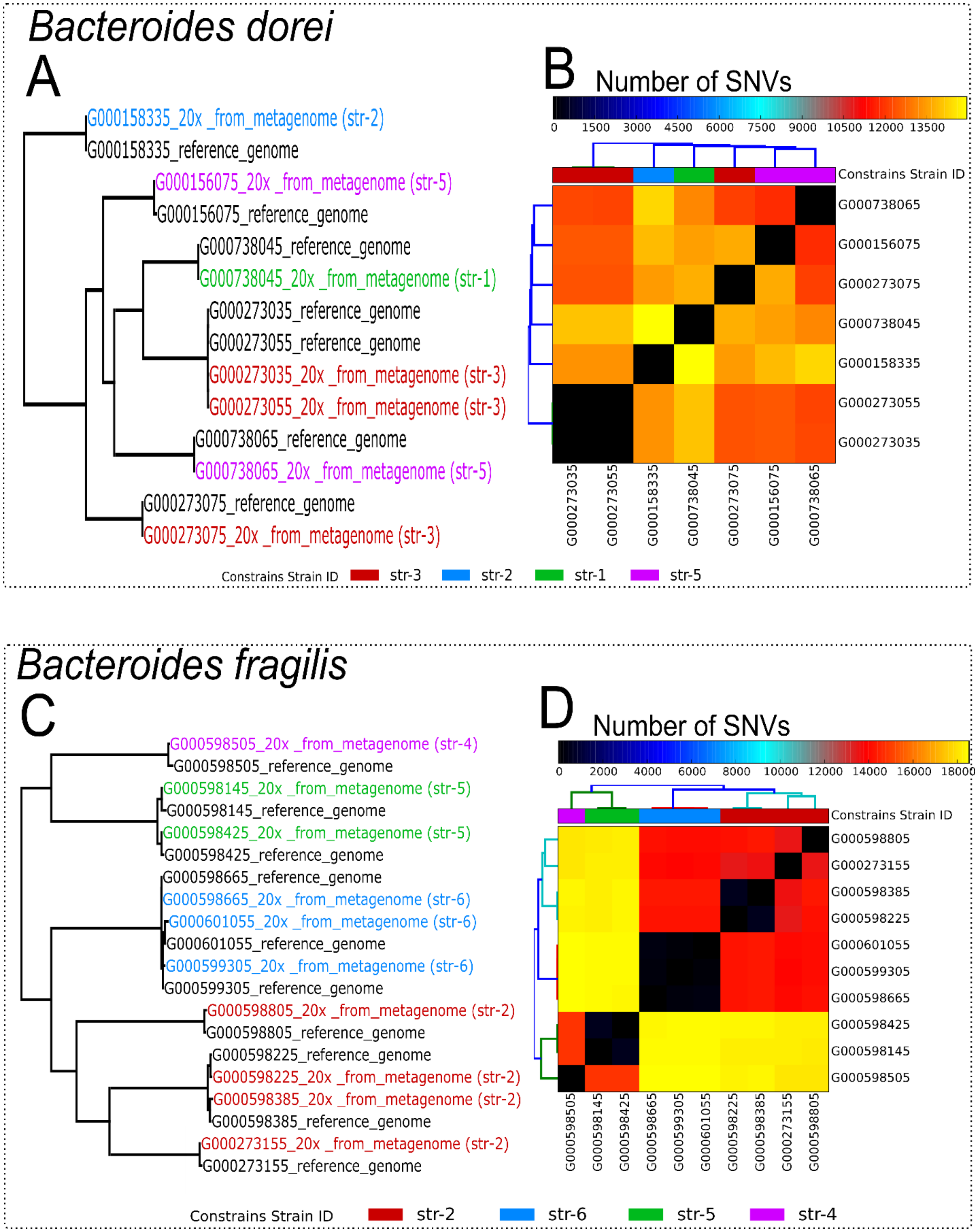

Supplemental Fig. S6. The comparison of the strains reconstructed by StrainPhlAn and ConStrains on the synthetic datasets of two species Bacteroides ovatus, and Bifidobacterium longum at 20x coverage. (A, C) The phylogenetic trees built from reference genomes and from corresponding synthetic samples by StrainPhlAn. In parenthesis, we report the strain IDs assigned by ConStrains. (B, D) The heatmap of SNV distances for the reference genomes on the whole core genome as obtained by the application of Prokka (5) and Roary (6) with the corresponding ConStrains strain ID.

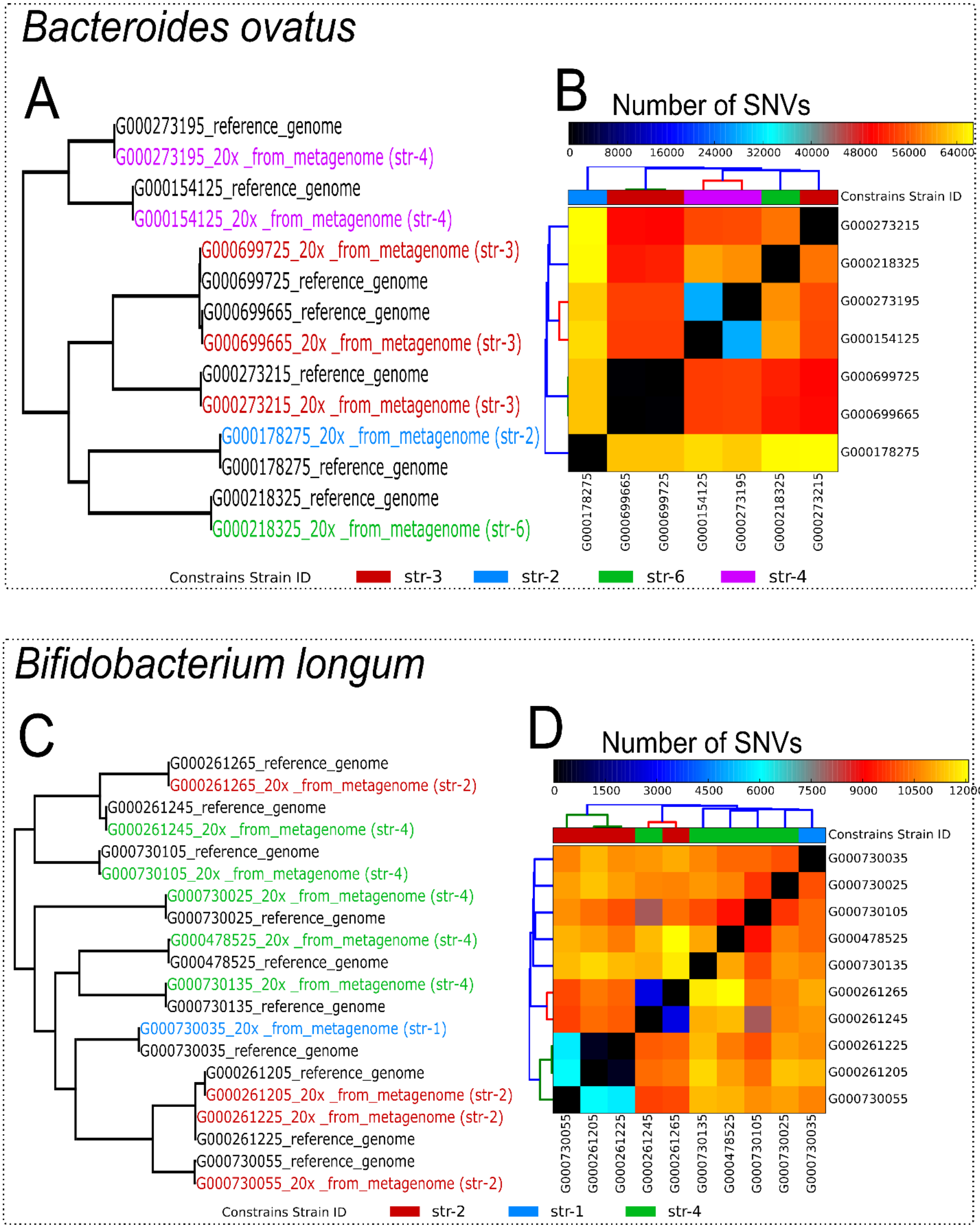

Supplemental Fig. S7. Comparison of the *B. animalis* strains reconstructed by StrainPhlAn from real sample with the reference genomes. We report the number of SNVs for each reconstruction and the SNV rates between the strains and the reference genomes of *B. animalis* subsp. lactis CNCM I-2494. On the right, we report the abundance and coverage of *B. animalis* in each sample.

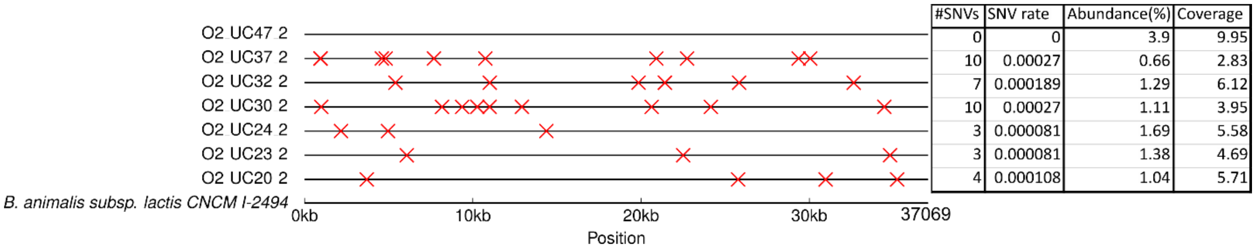

#SNVs	SNV rate	Abundance(%)	Coverage
0	0	3.9	9.95
10	0.00027	0.66	2.83
7	0.000189	1.29	6.12
10	0.00027	1.11	3.95
3	0.000081	1.69	5.58
3	0.000081	1.38	4.69
4	0.000108	1.04	5.71

Supplemental Fig. S8. StrainPhlAn reconstructions of *B. animalis* strains from metagenomes is consistent with the corresponding sequenced genomes. Our methodology reconstructed the marker genes of the *B. animalis*-based probiotic product taken by 19 subjects (4), when the strains were present at 2x coverage or higher (7 in the full set of 19). The phylogenetic placement of the seven *B. animalis* strains (red circles) are in accordance with the genomes of the probiotic product available in isolation (green and blue circles).

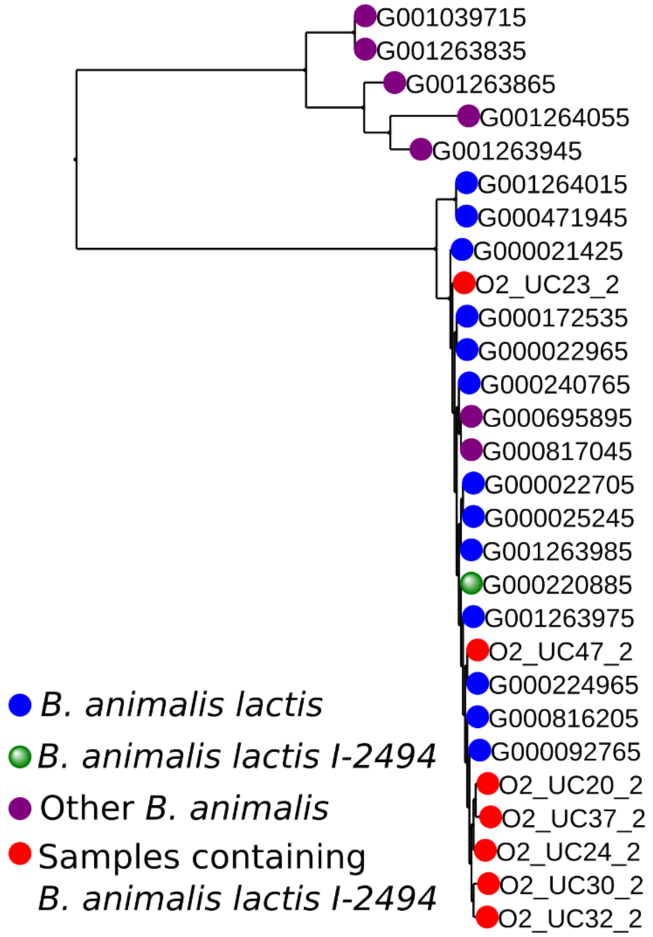

Supplemental Fig. S9. Distribution of non-polymorphic site prevalence in samples for the 40 most prevalent gut bacterial species. Each point represents, for each sample-species pair, the fraction of reconstructed marker positions that are non-polymorphic. In parenthesis we quantify the percentage of strains with >99.9% of non-polymorphic sites. The fraction of non-polymorphic sites varies from sample to sample and from species to species.

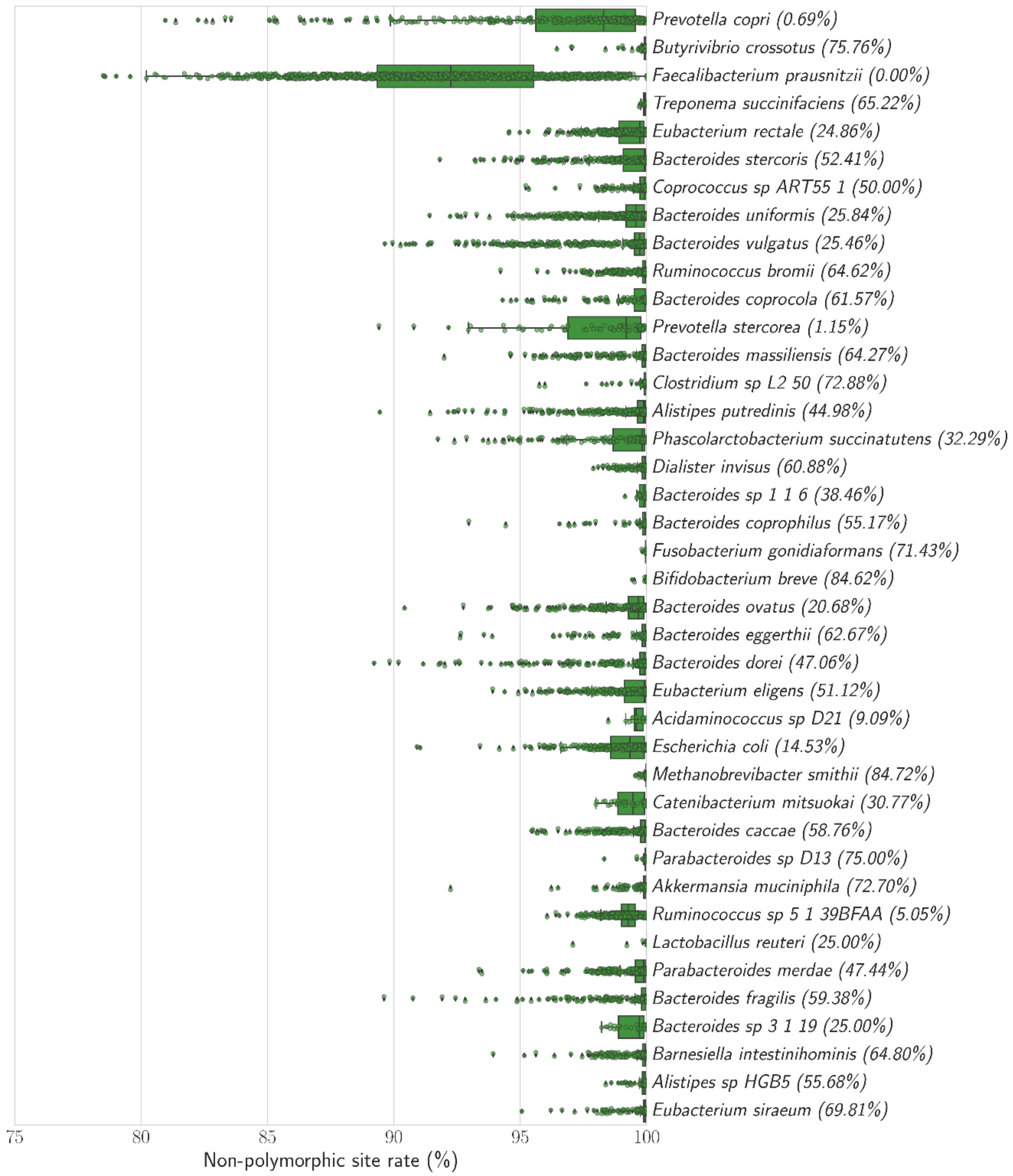

Supplemental Fig. S10. Rates of non-polymorphic positions and within-species dominant strain dominance are not correlated with relative abundance of the species. We contrasted here the non-polymorphic rates **(A)** and dominant strain frequency **(B)** against the relative abundance of the species the strain belongs to. Each point represent a different sample/species combination**.**

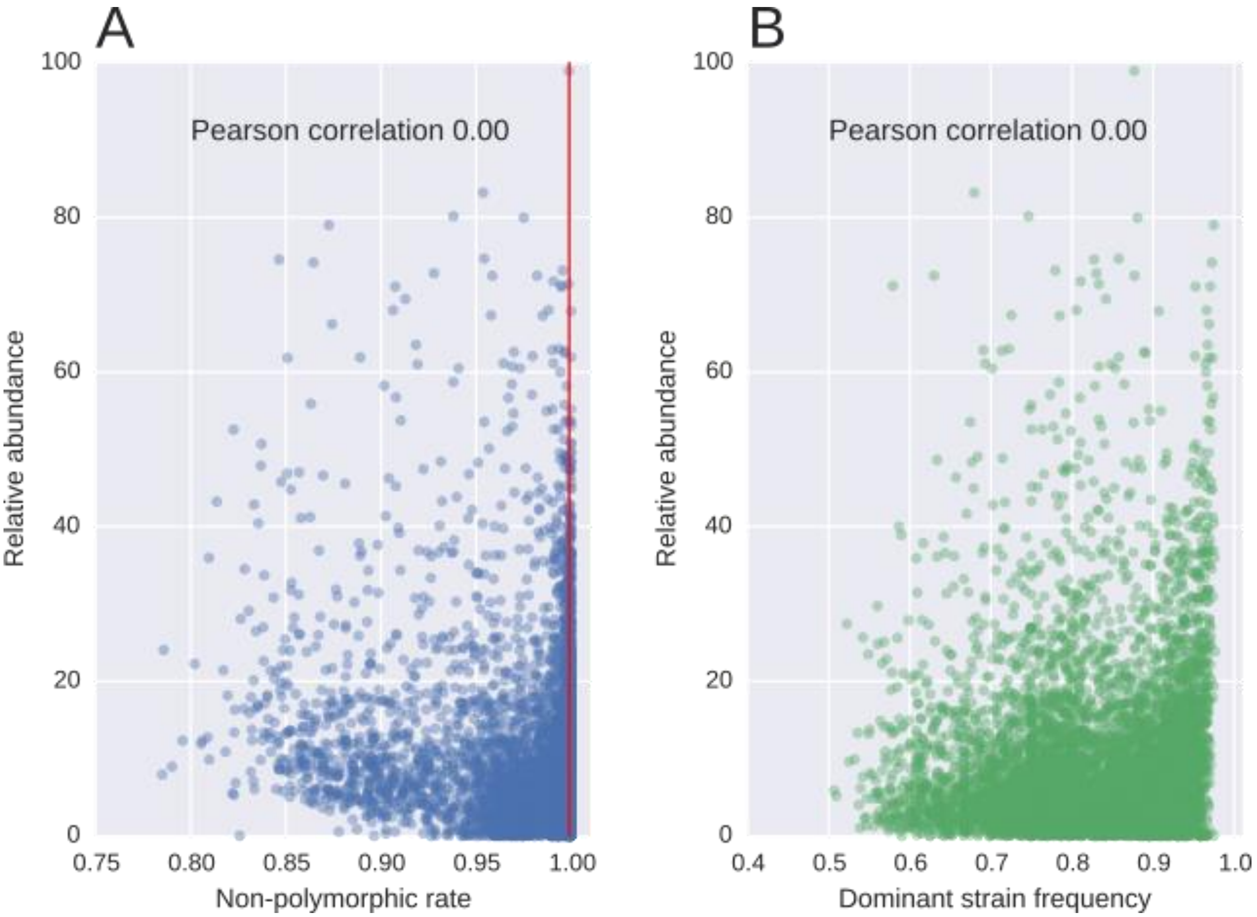

Supplemental Fig. S11. Strain retention rates and strain divergence in multiple longitudinal samples from the same subjects (131 from the HMP and 78 from MetaHIT). (A, C) The scatter plot of the retention rate versus the sampling interval between two samples of the same subject at two different time-points in the HMP and MetaHIT datasets respectively. For each subject, the retention rate is the proportion of species in which the subject harbors the same strain in the second time-point as the first. Two strains are considered to be the same if their normalized distance is less than $\mu_{intra\text{-}metahit} + 3\sigma_{intra\text{-}metahit}$ where $\mu_{intra\text{-}metahit}$ and $\sigma_{intra\text{-}metahit}$ are the median and standard deviation of the intra-metahit dominant distribution, respectively. (B, D) The scatter plot and histogram of the normalized distance versus sampling interval of two samples of the same subject at both time-points in the HMP and MetaHIT strains.

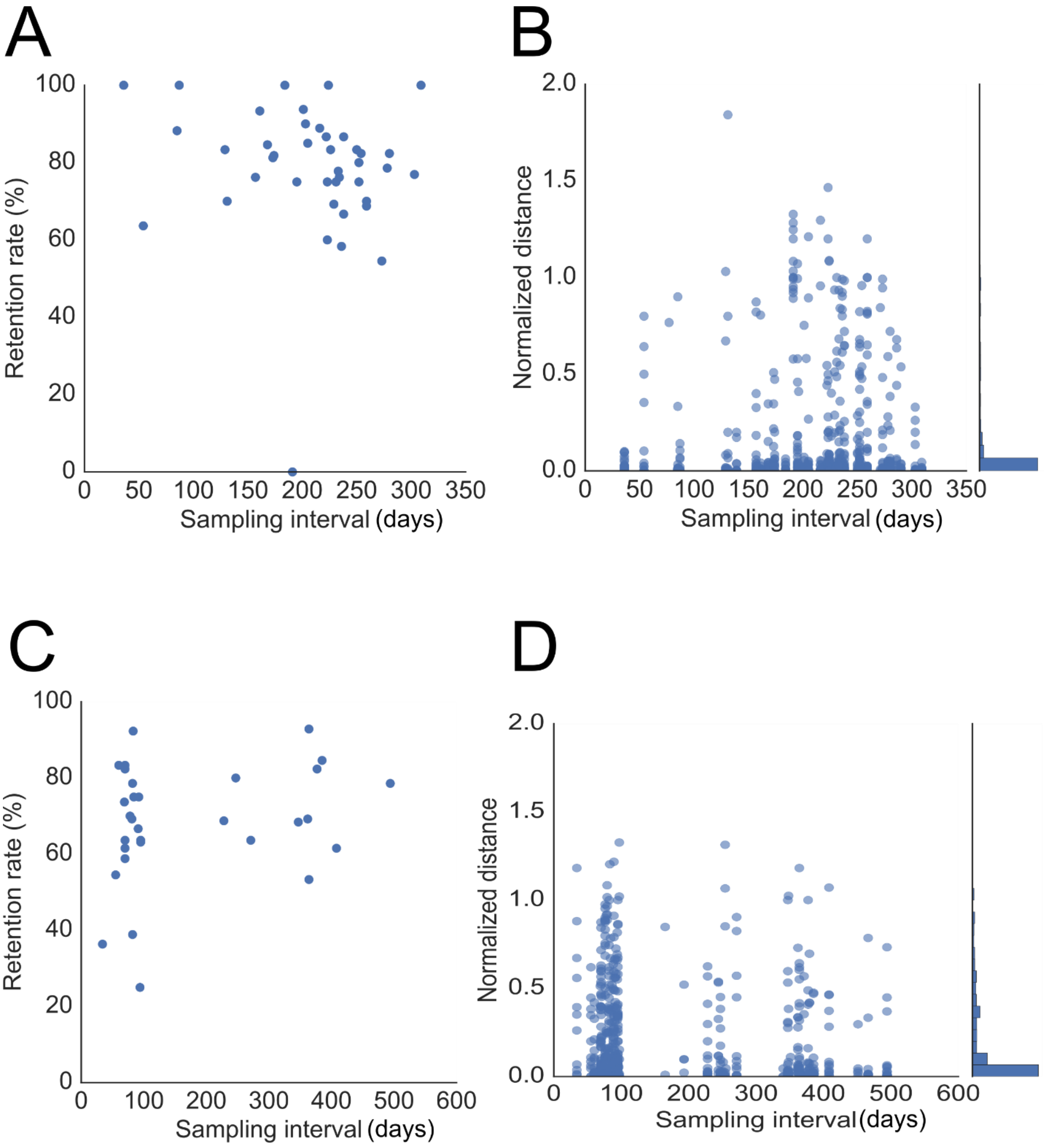

Supplemental Fig. S12. Population genomics structure of *Bacteroides coprocola* and their associated sampling countries. The strain-level relations are reported both as *the phylogeny* built on the concatenated alignments of each species-specific reconstructed markers (right) and as genetic distances measured on the same concatenated alignment and represented as the principal coordinate plot (left).

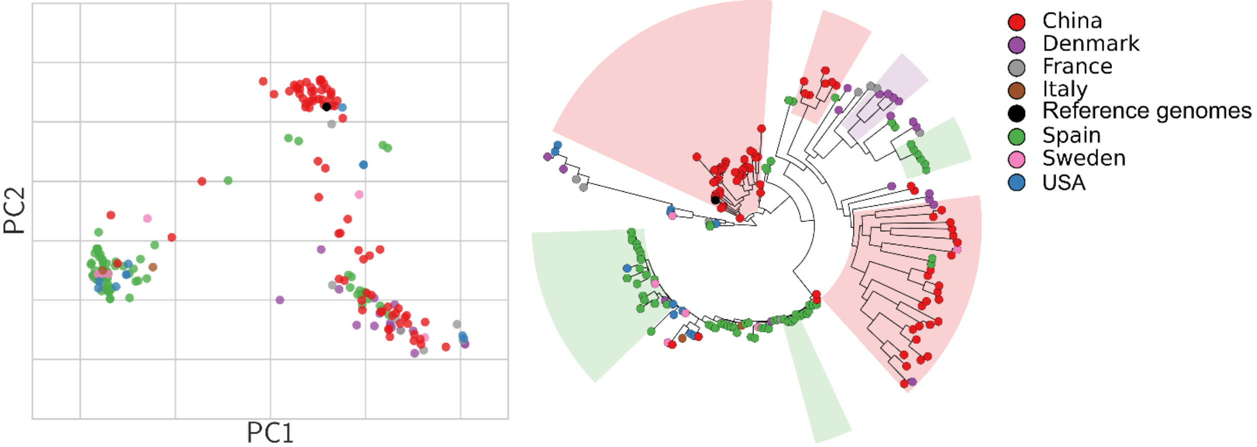

Supplemental Fig. S13. Population genomics structure of *Ruminococcus bromii* and their associated sampling countries. The strain-level relations are reported both as the phylogeny built on the concatenated alignments of each species-specific reconstructed markers (right) and as genetic distances measured on the same concatenated alignment and represented as the principal coordinate plot (left).

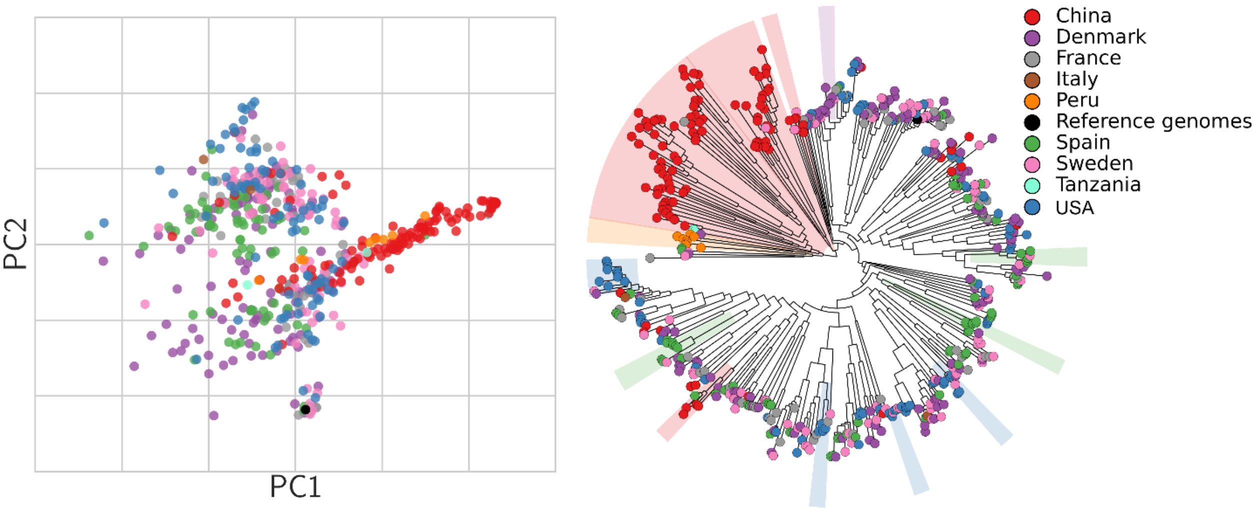

Supplemental Fig. S14. Population genomics structure of *Eubacterium eligens* and their associated sampling countries. The strain-level relations are reported both as the phylogeny built on the concatenated alignments of each species-specific reconstructed markers (right) and as genetic distances measured on the same concatenated alignment and represented as the principal coordinate plot (left).

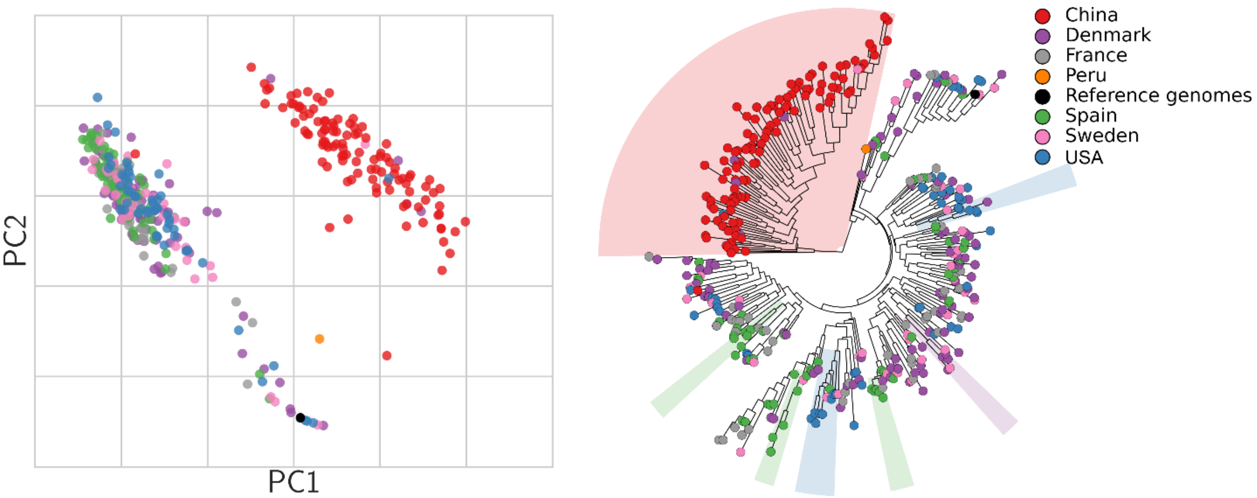

Supplemental Fig. S15. Population genomics structure of *Eubacterium hallii* and their associated sampling countries. The strain-level relations are reported both as the phylogeny built on the concatenated alignments of each species-specific reconstructed markers (right) and as genetic distances measured on the same concatenated alignment and represented as the principal coordinate plot (left).

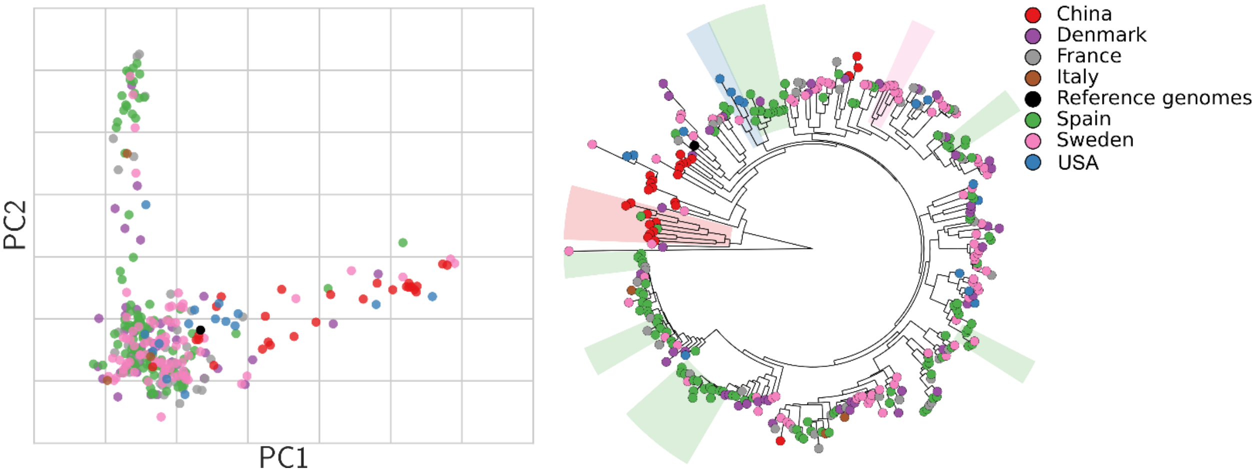

Supplemental Fig. S16. Population genomics structure of *Eubacterium siraeum* and their associated sampling countries. The strain-level relations are reported both as the phylogeny built on the concatenated alignments of each species-specific reconstructed markers (right) and as genetic distances measured on the same concatenated alignment and represented as the principal coordinate plot (left).

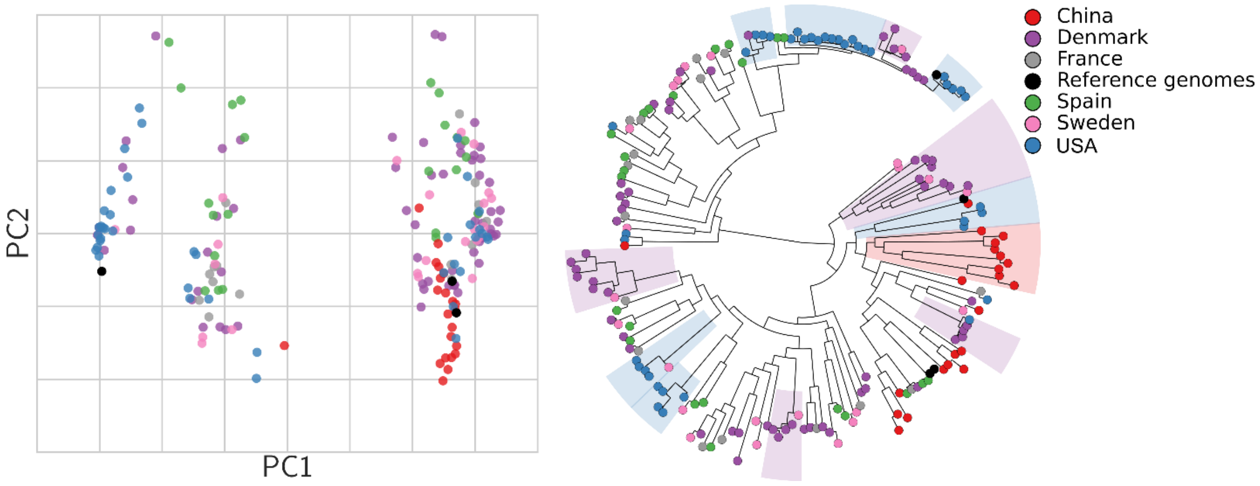

Supplemental Fig. S17. Genetic distances between strains in the same sub-clades (Intra-SCs) and between strains in different sub-clades (Inter-SC) for each of the forty most prevalent gut microbial species. The analysis reveals that the strains within a SC have very limited genetic diversity (generally lower than 0.1% SNP rates, whereas strains in different SC are from 5 to 10 times more genetically variable.

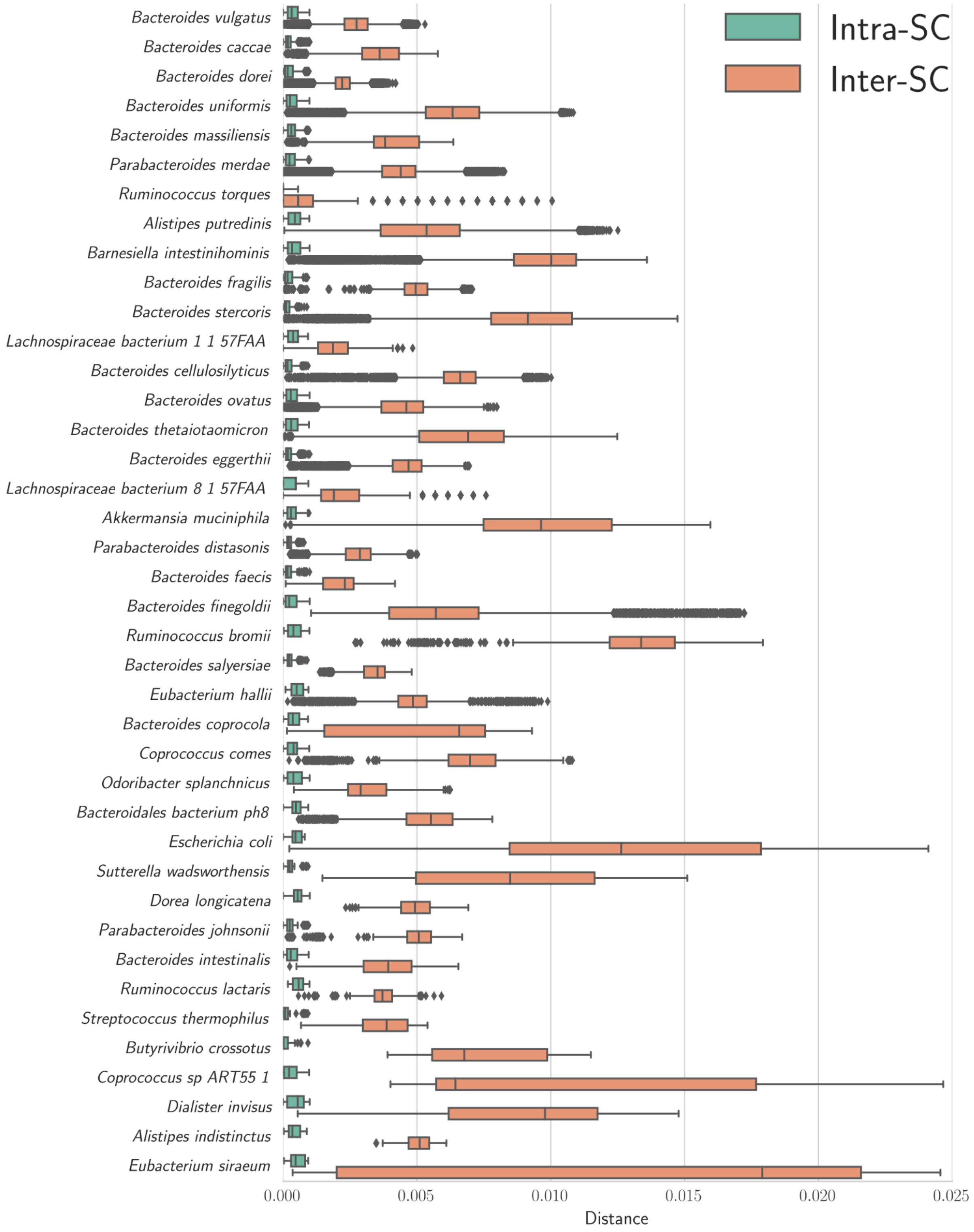

Supplemental Fig. S18. Population genomics structure of *Bacteroides intestinalis* and their associated sampling countries. The strain-level relations are reported both as the phylogeny built on the concatenated alignments of each species-specific reconstructed markers (right) and as genetic distances measured on the same concatenated alignment and represented as the principal coordinate plot (left).

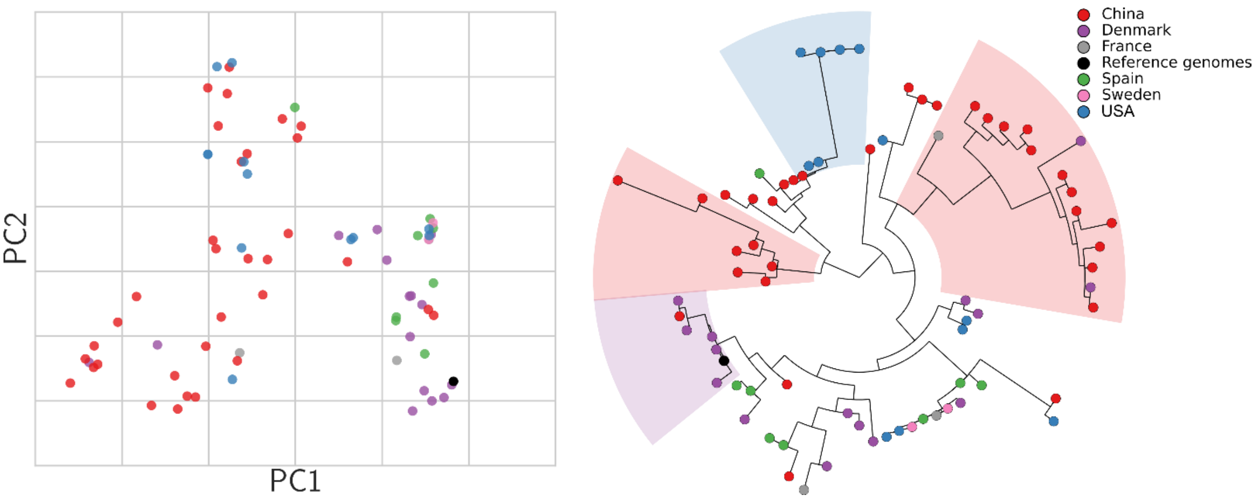

Supplemental Fig. S19. Population genomics structure of *Bacteroides massiliensis* and their associated sampling countries. The strain-level relations are reported both as the phylogeny built on the concatenated alignments of each species-specific reconstructed markers (right) and as genetic distances measured on the same concatenated alignment and represented as the principal coordinate plot (left).

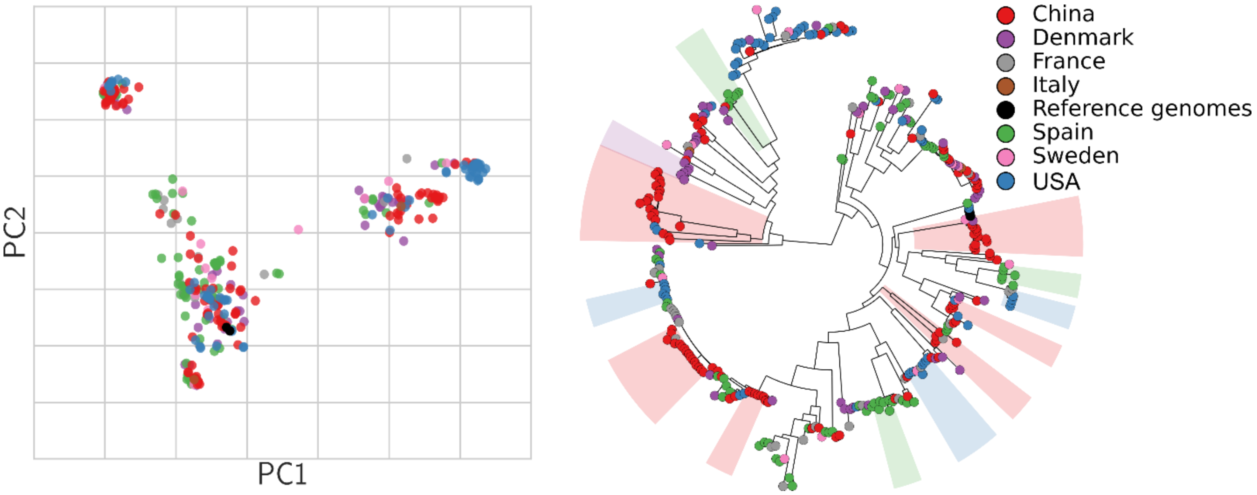

Supplemental Fig. S20. Population genomics structure of *Butyrivibrio crossotus* and their associated sampling countries. The strain-level relations are reported both as the phylogeny built on the concatenated alignments of each species-specific reconstructed markers (right) and as genetic distances measured on the same concatenated alignment and represented as the principal coordinate plot (left).

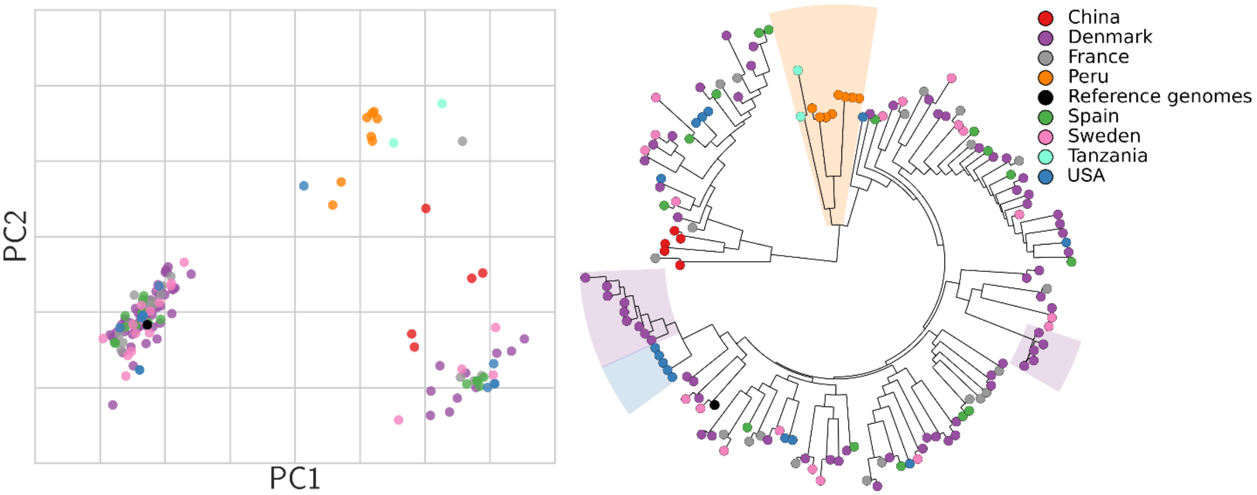

Supplemental Fig. S21. Population genomics structure of *Dialister invisus* and their associated sampling countries. The strain-level relations are reported both as the phylogeny built on the concatenated alignments of each species-specific reconstructed markers (right) and as genetic distances measured on the same concatenated alignment and represented as the principal coordinate plot (left).

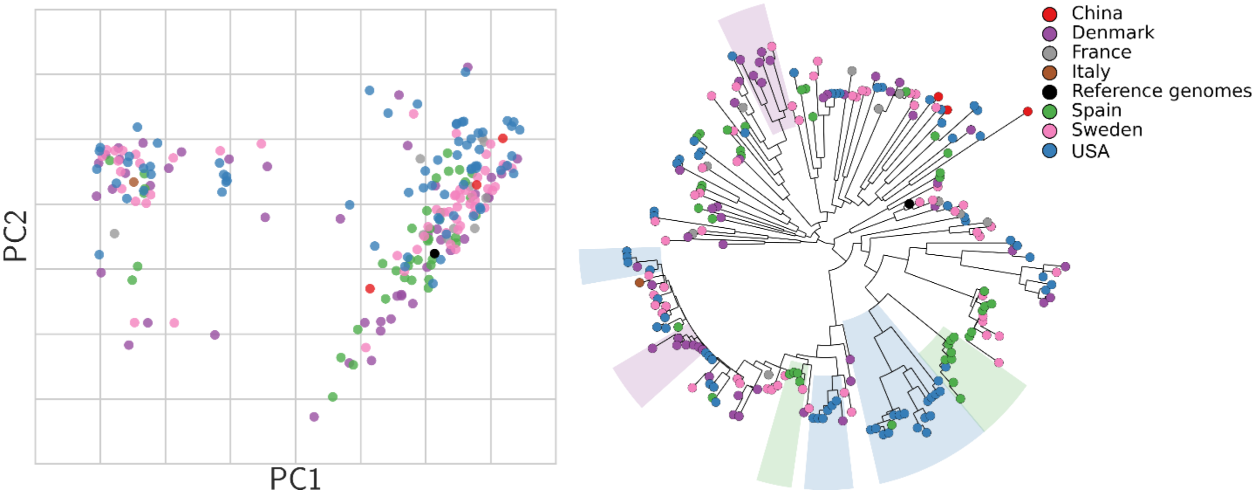

Supplemental Fig. S22. **Population genomics structure of *Dorea formicigenerans* and their associated sampling countries.** The strain-level relations are reported both as the phylogeny built on the concatenated alignments of each species-specific reconstructed markers (right) and as genetic distances measured on the same concatenated alignment and represented as the principal coordinate plot (left).

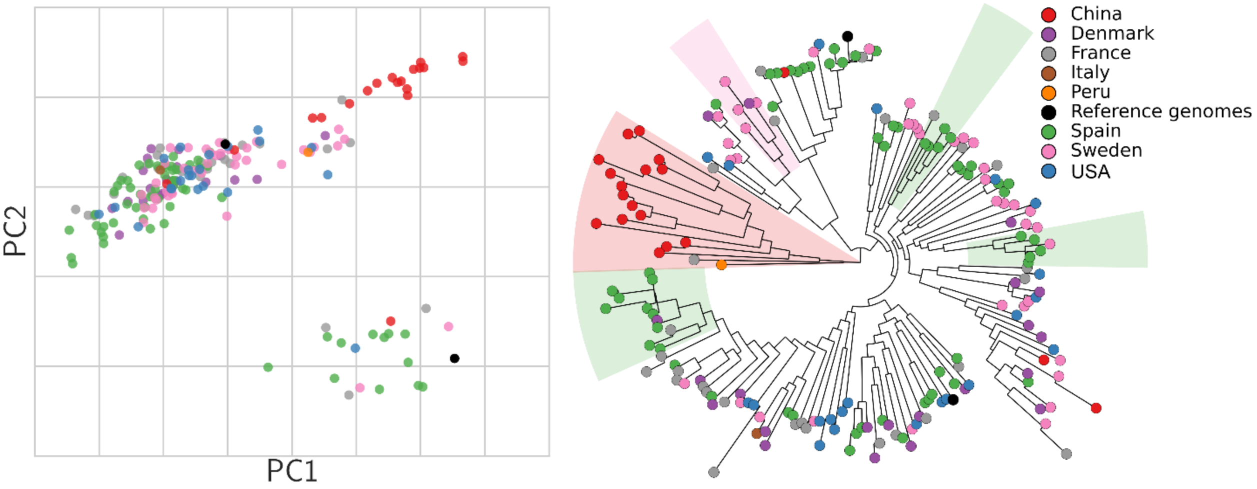

Supplemental Fig. S23. Population genomics structure of Prevotella stercorea and their associated sampling countries. The strain-level relations are reported both as the phylogeny built on the concatenated alignments of each species-specific reconstructed markers (right) and as genetic distances measured on the same concatenated alignment and represented as the principal coordinate plot (left).

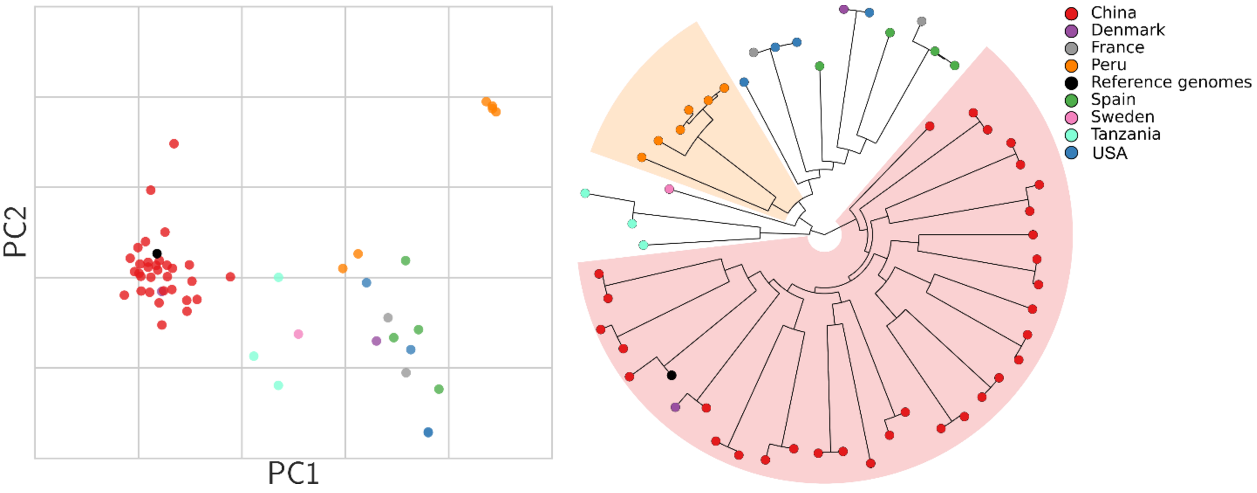

Supplemental Fig. S24. Population genomics structure of *Ruminococcus lactaris* and their associated sampling countries. The strain-level relations are reported both as the phylogeny built on the concatenated alignments of each species-specific reconstructed markers (right) and as genetic distances measured on the same concatenated alignment and represented as the principal coordinate plot (left).

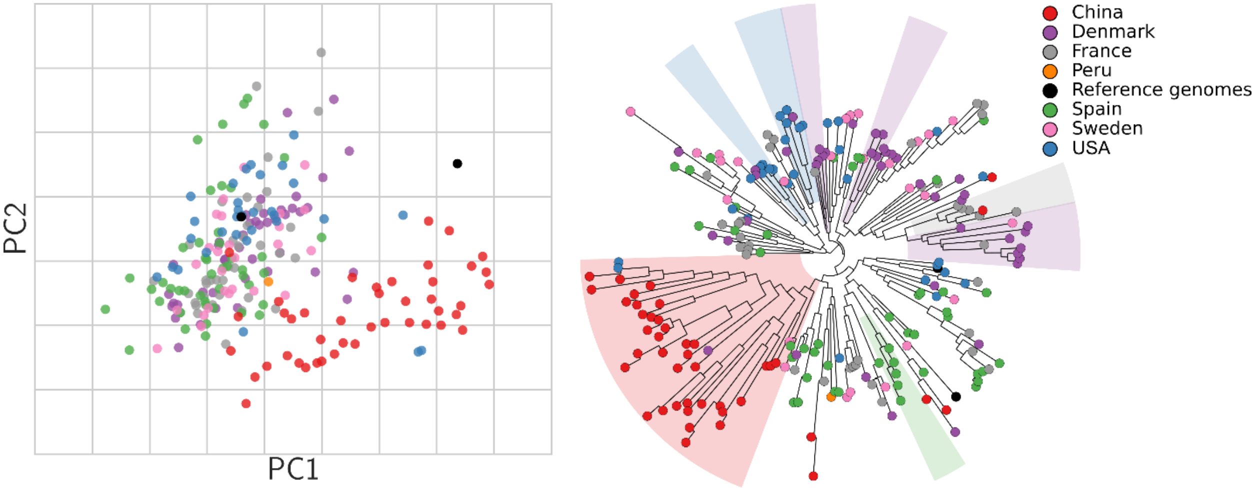

Supplemental Fig. S25. The phylogenetic tree of *Bacteroides caccae* with the identified subspecies-subclades (SC) and their associated sampling countries. Percentages refer to the prevalence of the dominant country in each SC.

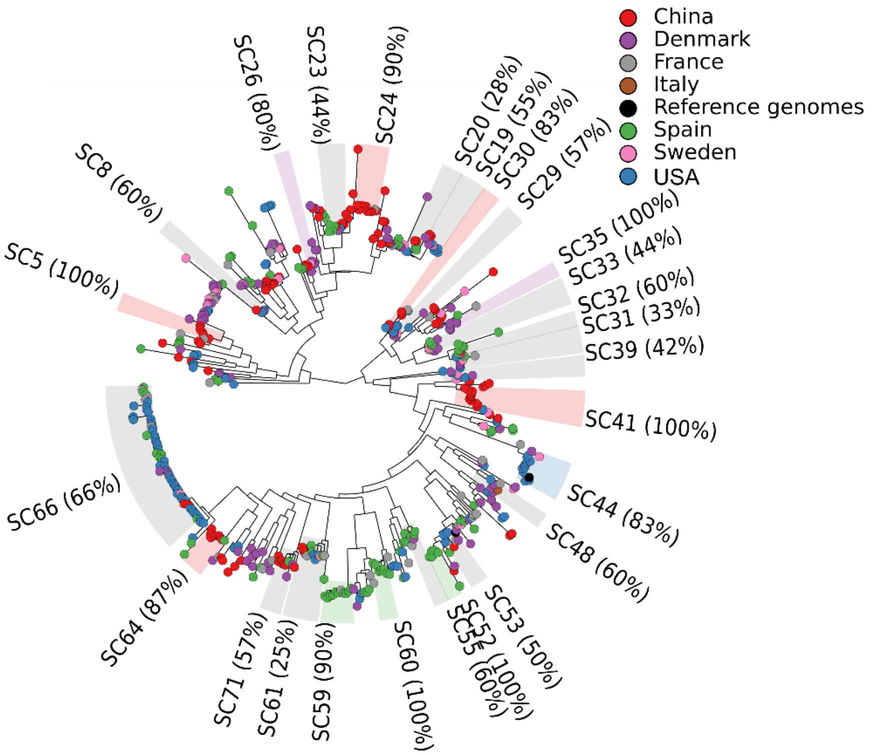

Supplemental Fig. S26. The phylogentic tree of *Akkermansia muciniphila* with the identified subspecies-subclades (SC) and their associated sampling countries. Percentages refer to the prevalence of the dominant country in each SC.

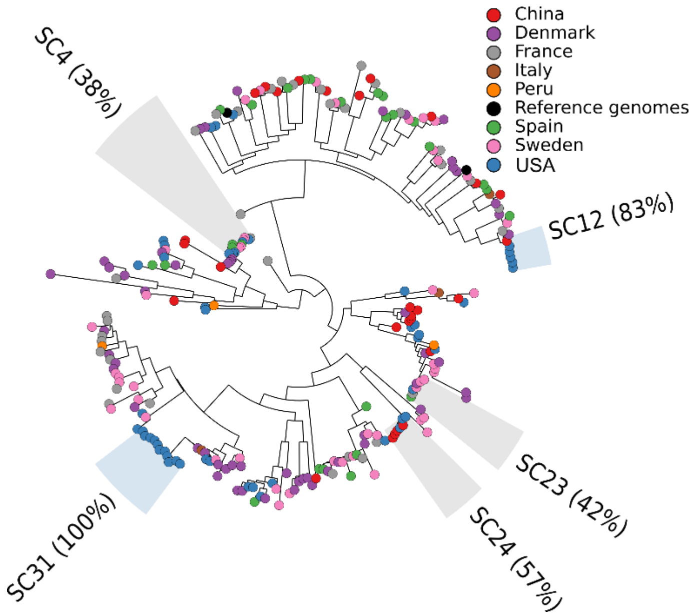

Supplemental Fig. S27. The phylogenetic tree of *Bacteroides cellulosilyticus* with the identified subspecies-subclades (SC) and their associated sampling countries. Percentages refer to the prevalence of the dominant country in each SC.

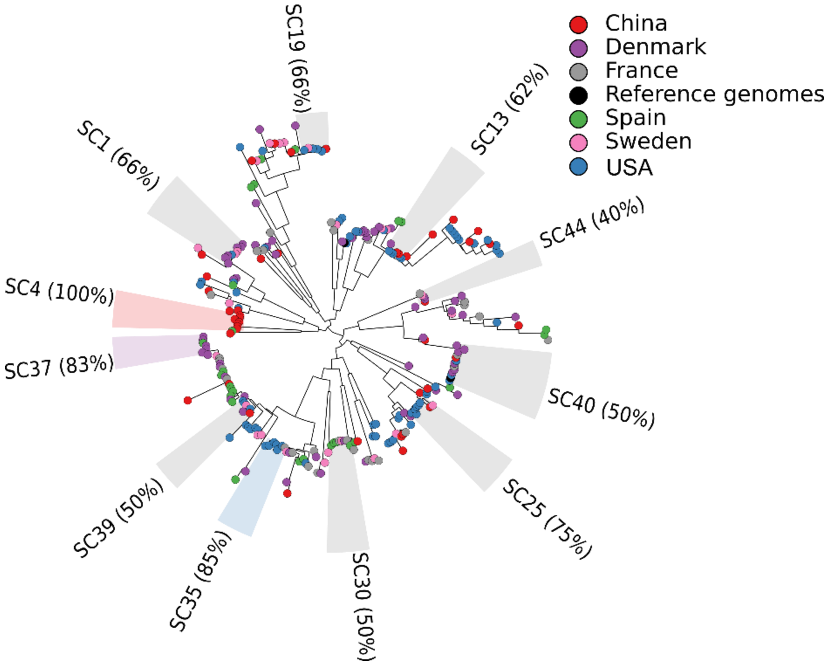

Supplemental Fig. S28. The phylogenetic tree of *Bacteroides coprocola* with the identified subspecies-subclades (SC) and their associated sampling countries. Percentages refer to the prevalence of the dominant country in each SC.

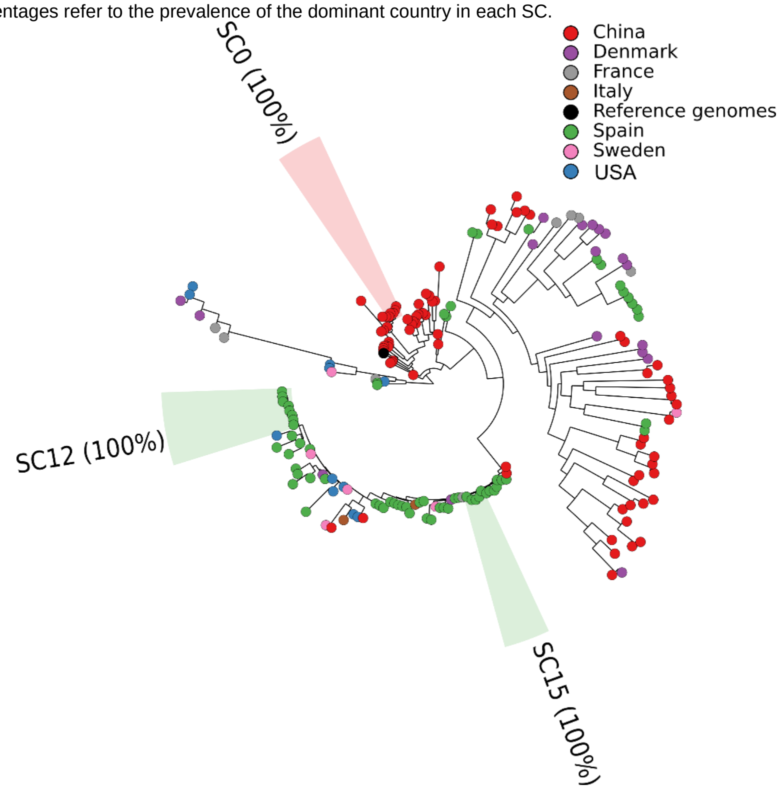

Supplemental Fig. S29. The phylogenetic tree of *Bacteroides dorei* with the identified subspecies-subclades (SC) and their associated sampling countries. Percentages refer to the prevalence of the dominant country in each SC.

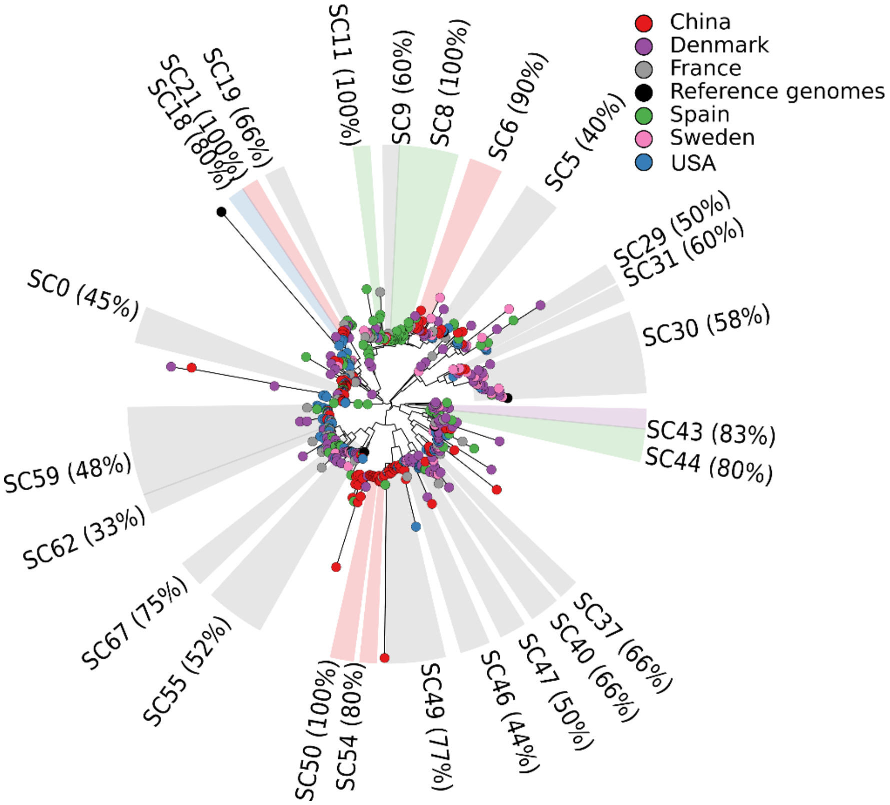

Supplemental Fig. S30. The phylogenetic tree of *Bacteroides eggerthii* with the identified subspecies-subclades (SC) and their associated sampling countries. Percentages refer to the prevalence of the dominant country in each SC.

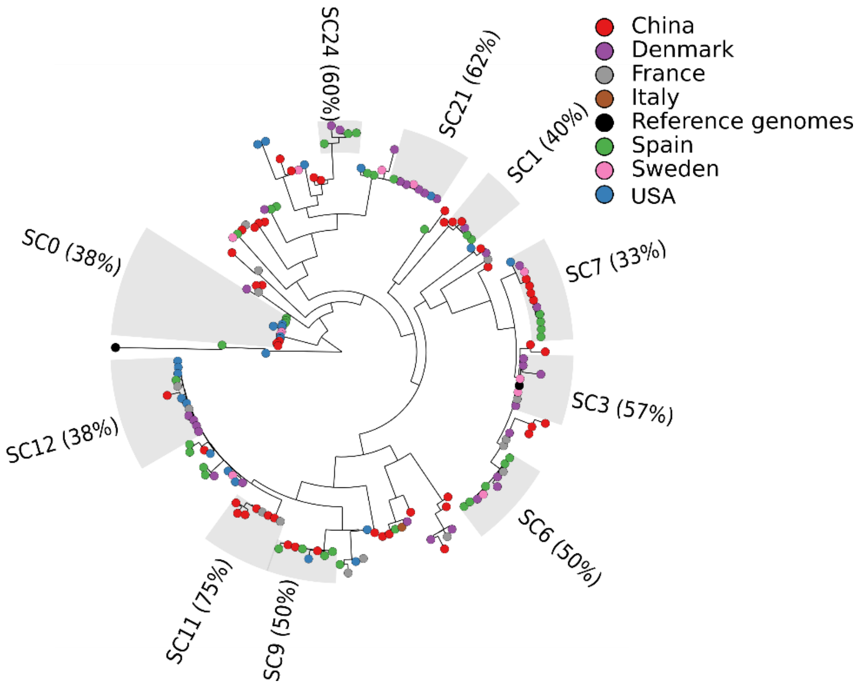

Supplemental Fig. S31. The phylogenetic tree of *Bacteroides faecis* with the identified subspecies-subclades (SC) and their associated sampling countries. Percentages refer to the prevalence of the dominant country in each SC.

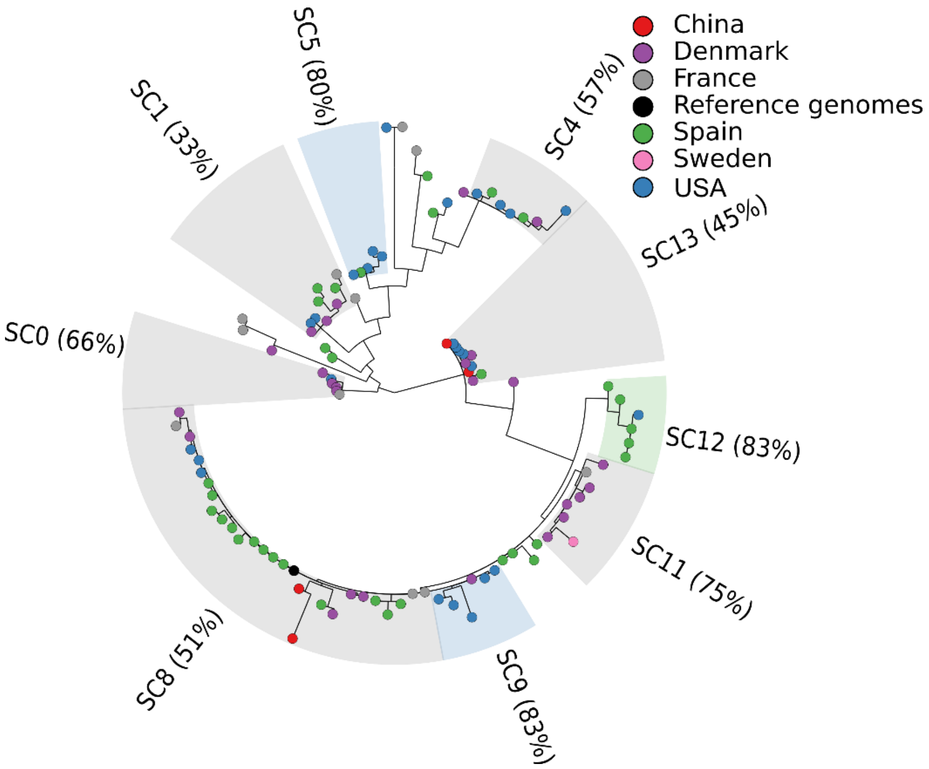

Supplemental Fig. S32. The phylogenetic tree of *Bacteroides finegoldii* with the identified subspecies-subclades (SC) and their associated sampling countries. Percentages refer to the prevalence of the dominant country in each SC.

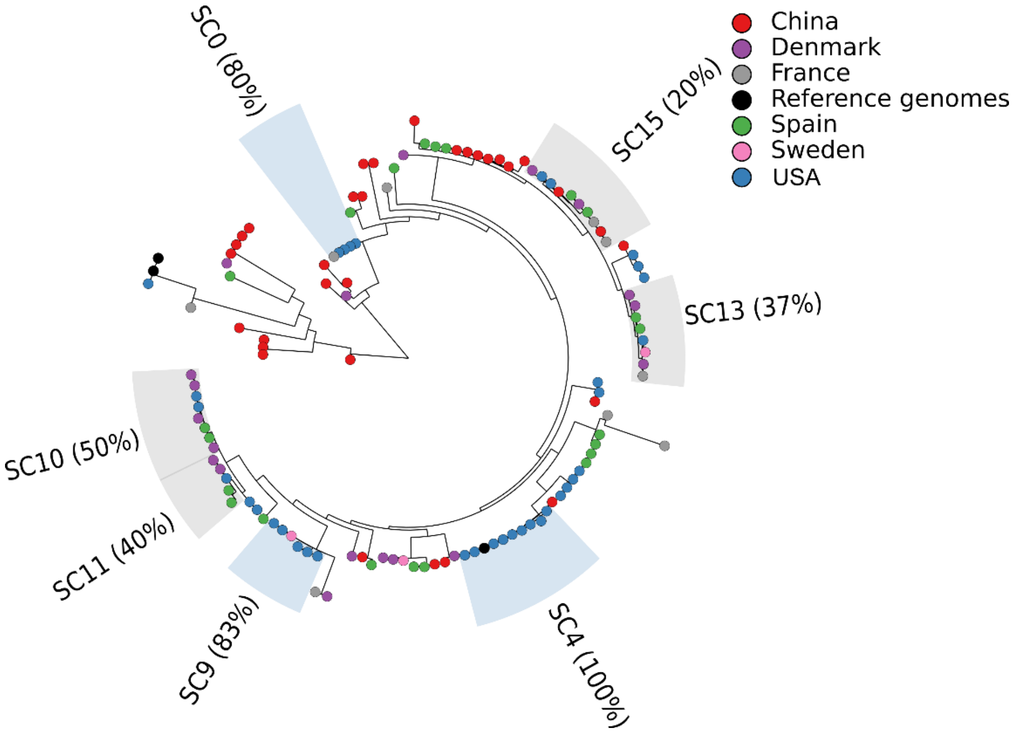

Supplemental Fig. S33. The phylogenetic tree of *Bacteroides massiliensis* with the identified subspecies-subclades (SC) and their associated sampling countries. Percentages refer to the prevalence of the dominant country in each SC.

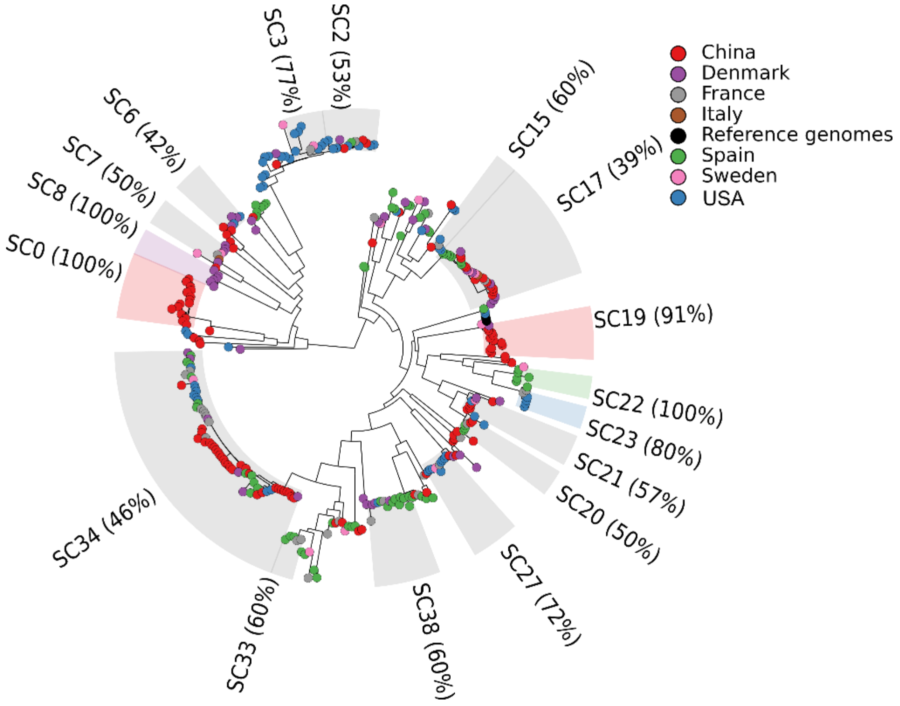

Supplemental Fig. S34. The phylogenetic tree of *Bacteroides ovatus* with the identified subspecies-subclades (SC) and their associated sampling countries. Percentages refer to the prevalence of the dominant country in each SC.

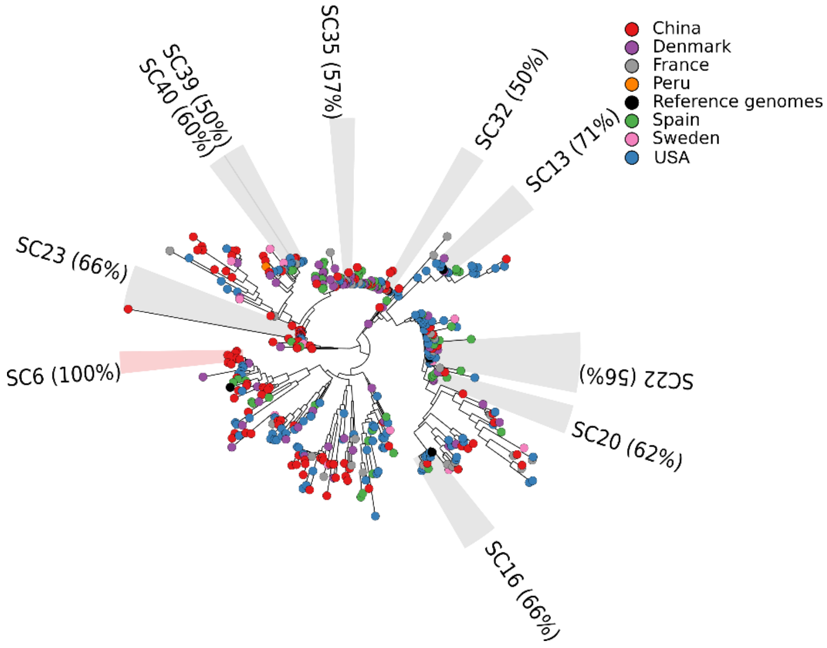

Supplemental Fig. S35. The phylogenetic tree of *Bacteroides salyersiae* with the identified subspecies-subclades (SC) and their associated sampling countries. Percentages refer to the prevalence of the dominant country in each SC.

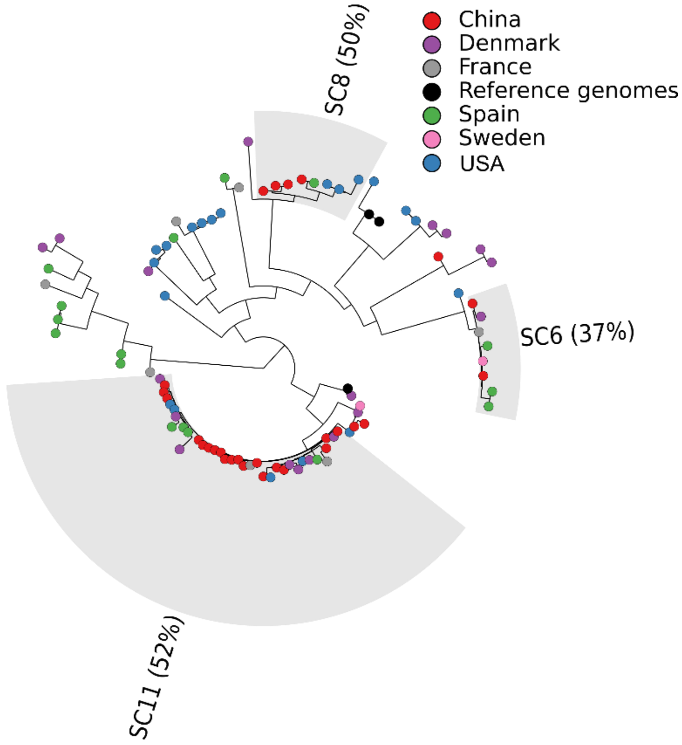

Supplemental Fig. S36. The phylogenetic tree of *Bacteroides stercoris* with the identified subspecies-subclades (SC) and their associated sampling countries. Percentages refer to the prevalence of the dominant country in each SC.

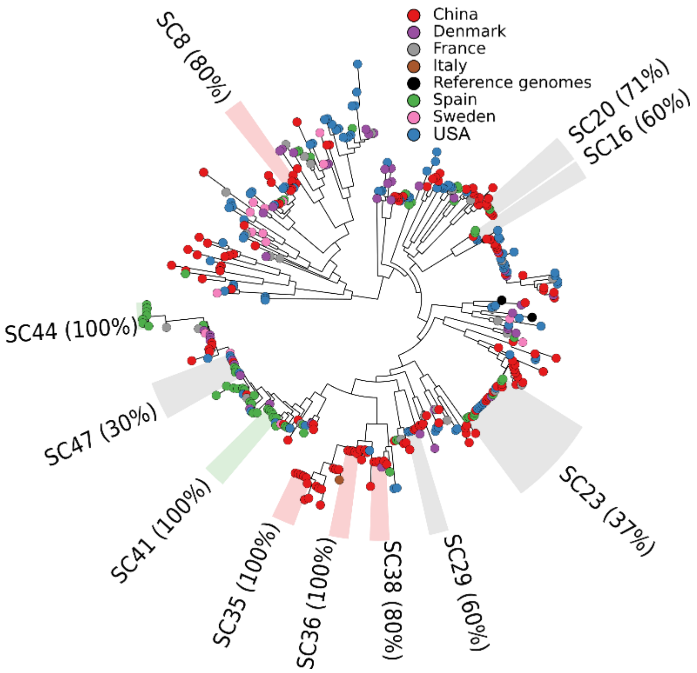

Supplemental Fig. S37. The phylogenetic tree of *Lachnospiraceae bacterium 1_1_57FAA* with the identified subspecies-subclades (SC) and their associated sampling countries. Percentages refer to the prevalence of the dominant country in each SC.

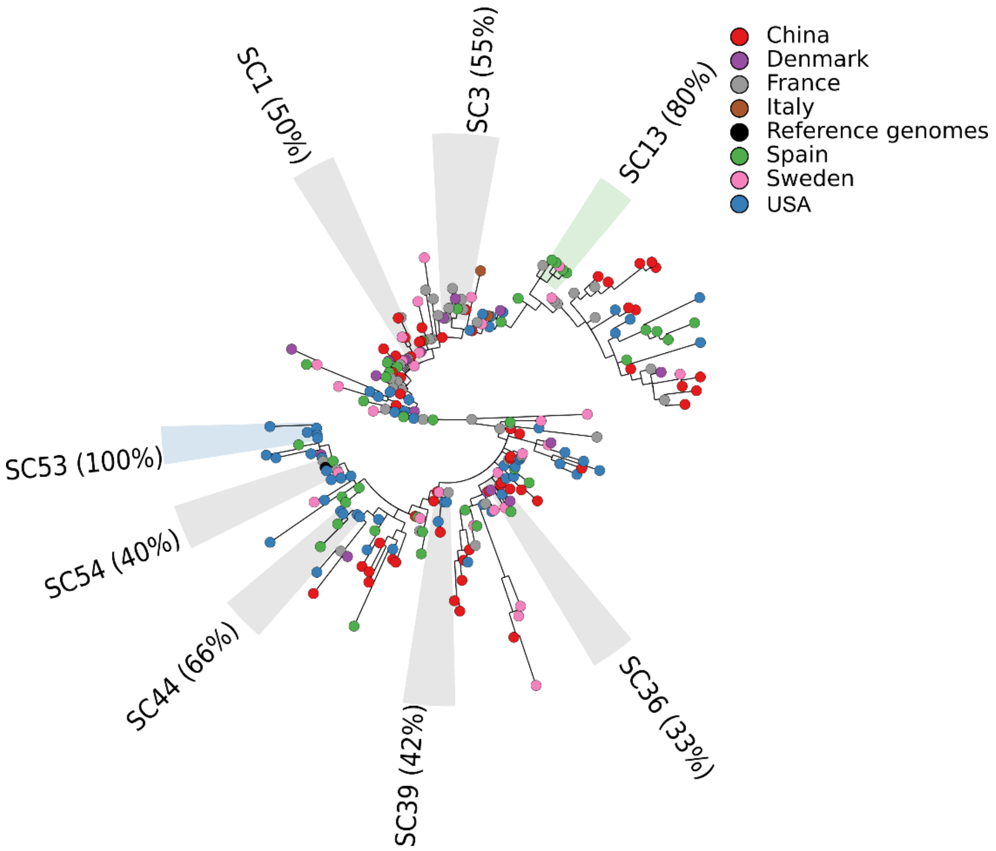

Supplemental Fig. S38. The phylogenetic tree of *Parabacteroides distasonis* with the identified subspecies-subclades (SC) and their associated sampling countries. Percentages refer to the prevalence of the dominant country in each SC.

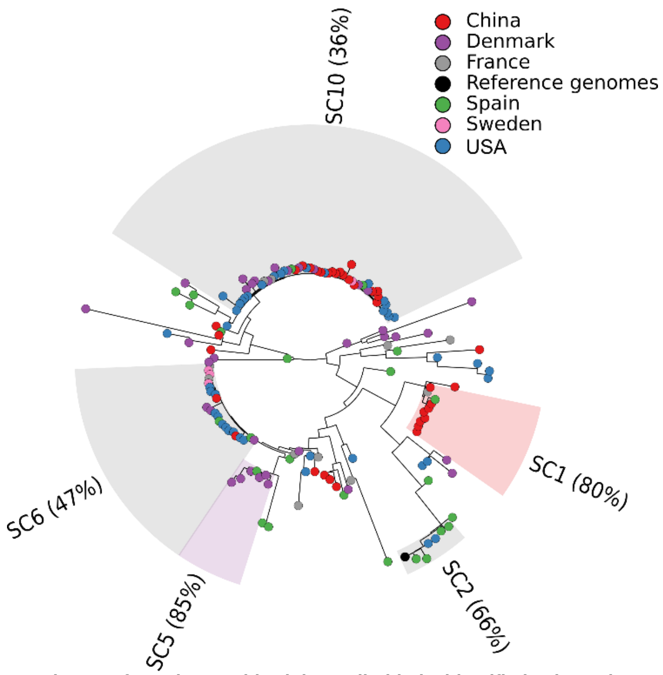

Supplemental Fig. S39. The phylogenetic tree of *Parabacteroides johnsonii* with the identified subspecies-subclades (SC) and their associated sampling countries. Percentages refer to the prevalence of the dominant country in each SC.

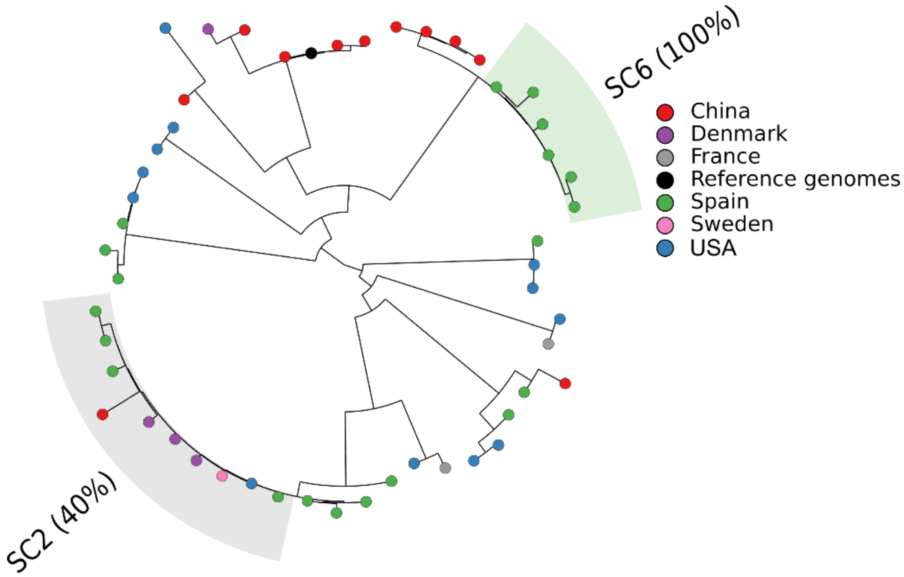

Supplemental Fig. S40. The phylogenetic tree of *Streptococcus thermophiles* with the identified subspecies-subclades (SC) and their associated sampling countries. Percentages refer to the prevalence of the dominant country in each SC.

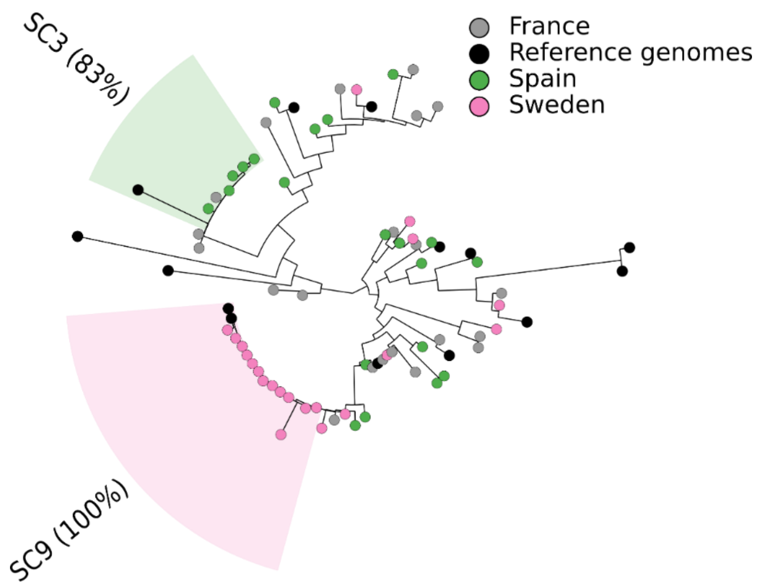

Supplemental Fig. S41. The phylogenetic tree of *Sutterella wadsworthensis* with the identified subspecies-subclades (SC) and their associated sampling countries. Percentages refer to the prevalence of the dominant country in each SC.

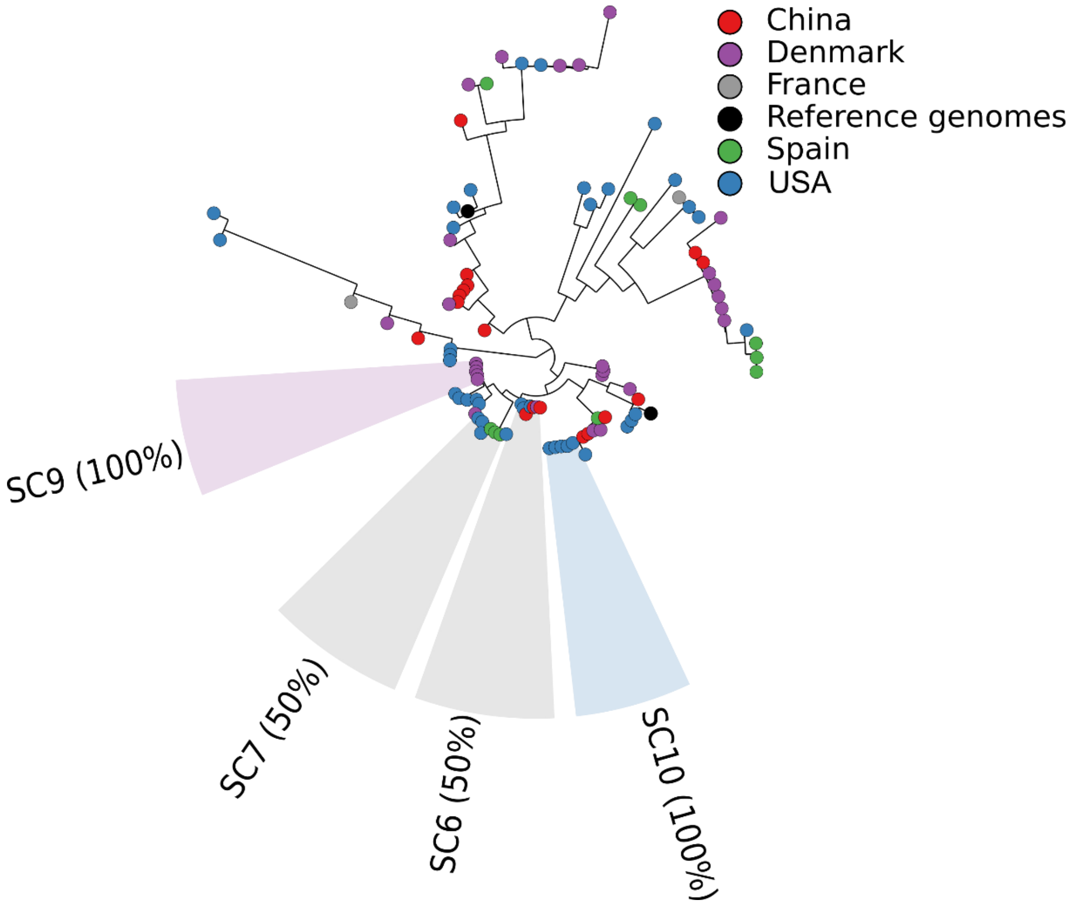

Supplemental Fig. S42. Subspecies-clades identified for the most prevalent species within the whole sample set of 1,590 metagenomes and their geographical association. We report here a subset of 125 species, the others are shown in Supplemental Fig. S43 and S44. For each species, we report first the pie-chart of the overall prevalence for each country, and then the largest country-specific subtrees ordered by size. The (few) subtrees containing available reference isolate genomes are marked with a black border.

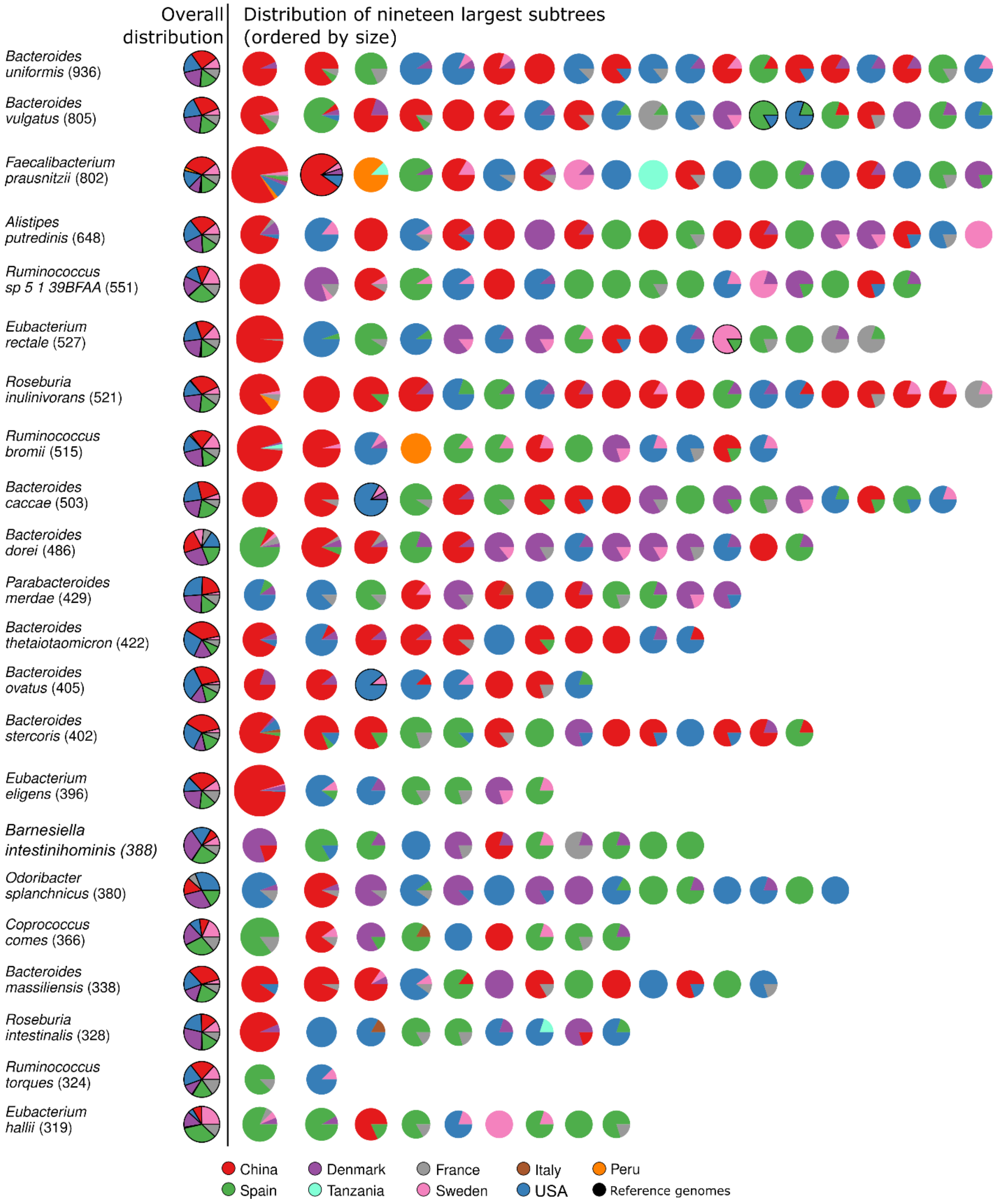

Supplemental Fig. S43. Subspecies-clades identified for the most prevalent species within the whole sample set of 1,590 metagenomes and their geographical association. We report here a subset of 125 species, the others are shown in Supplemental Fig. S42 and S44. For each species, we report first the pie-chart of the overall prevalence for each country, and then the largest country-specific subtrees ordered by size. The (few) subtrees containing available reference isolate genomes are marked with a black border.

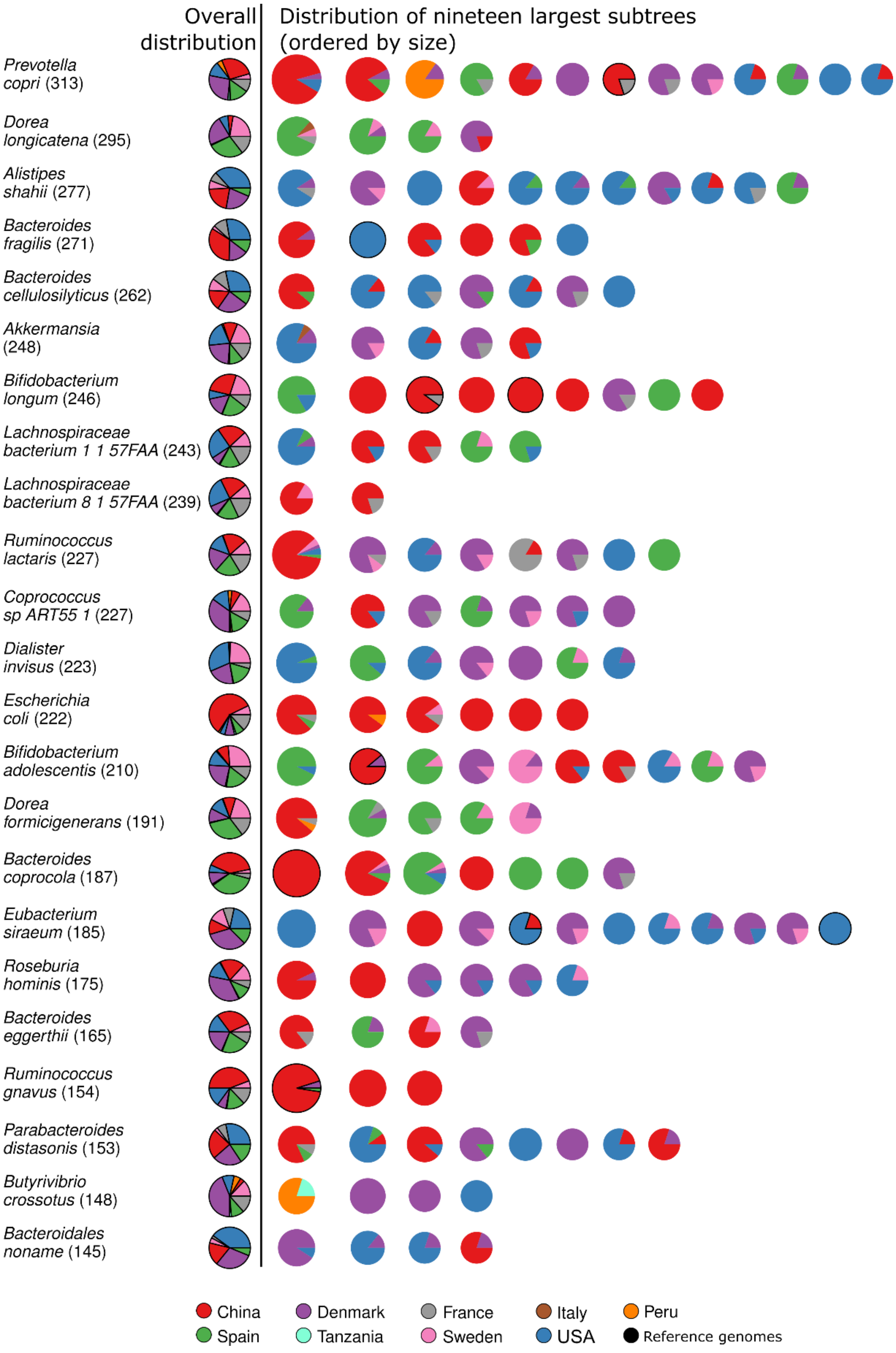

Supplemental Fig. S44. Subspecies-clades identified for the most prevalent species within the whole sample set of 1,590 metagenomes and their geographical association. We report here a subset of 125 species, the others are shown in Supplemental Fig. S42 and S43. For each species, we report first the pie-chart of the overall prevalence for each country, and then the largest country-specific subtrees ordered by size. The (few) subtrees containing available reference isolate genomes are marked with a black border.

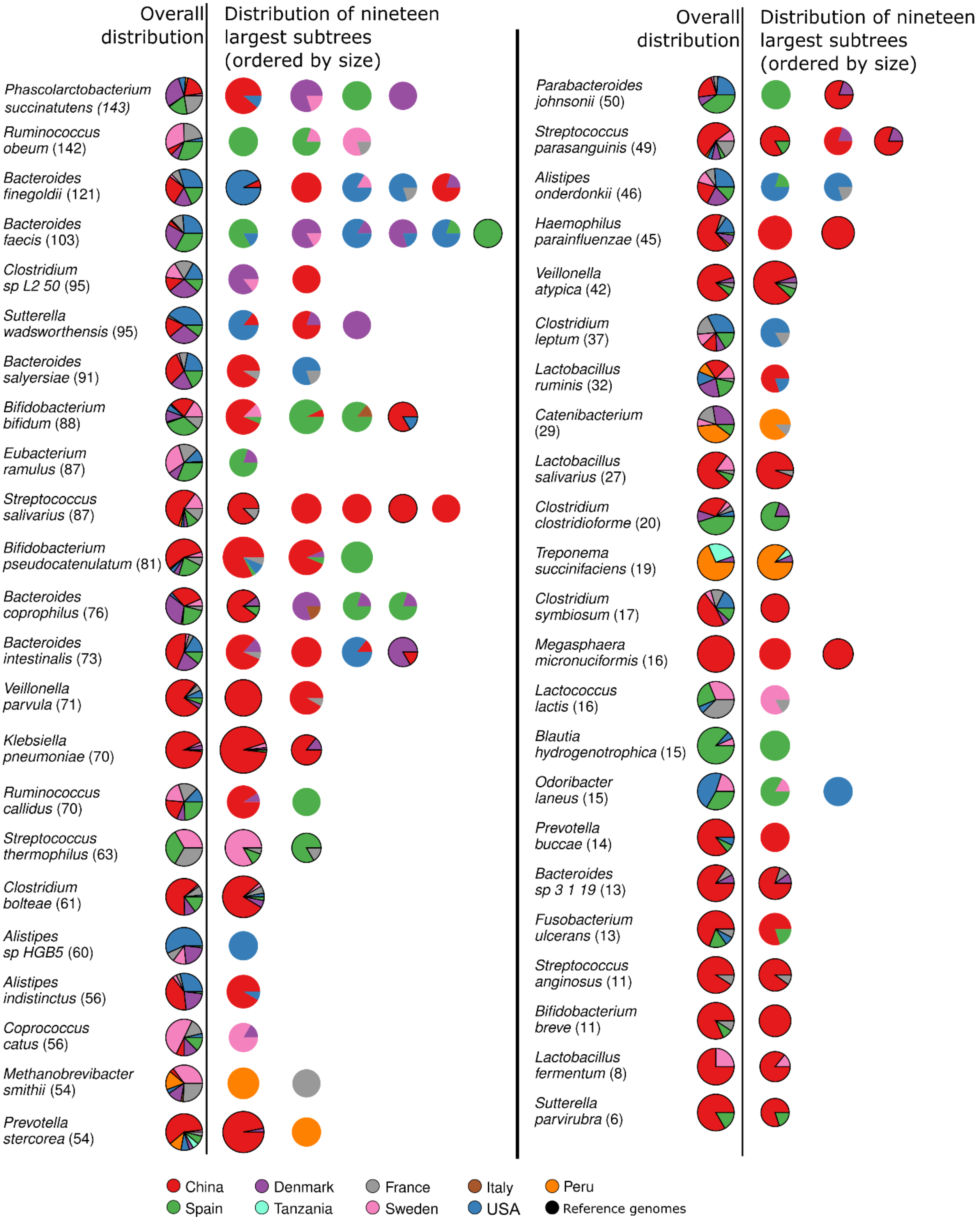

Supplemental Fig. S45. The genetic diversity of different species. For each species, we compute the number of samples in which the species is present (denoted as "Total prevalence"), and the relative genetic variability (measured by SNV rate median) of the strains in these samples. The bubble diameter is proportional to the average abundance and the opacity is scaled up with the sequence length. In the legend, for each genus, we present also the number of plotted species in that genus. The species diversity varies significantly from 0.000179 (*Bifidobacterium animalis*) to 0.038977 (*Phascolarctobacterium succinatutens*). Similarly, the number of samples having a species also alters notably from 14 (*Bifidobacterium animalis*) to 958 (*Bacteroides uniformis*). In other words, some species appear universally whereas the others are subject-dependent.

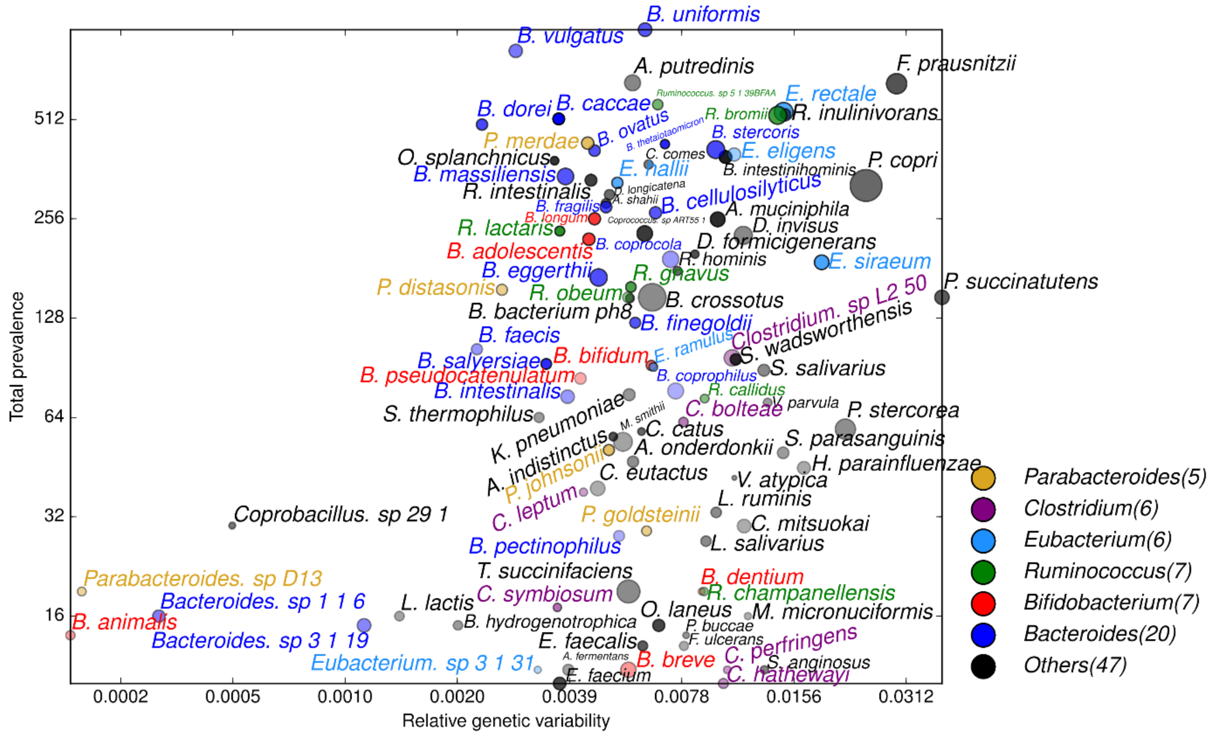

Supplemental Fig. S46. Fraction of total branch length spanned by strains sequenced in isolation (reference genomes) versus total branch length spanned by strains retrieved from metagenomes of species with at least three reference genomes.

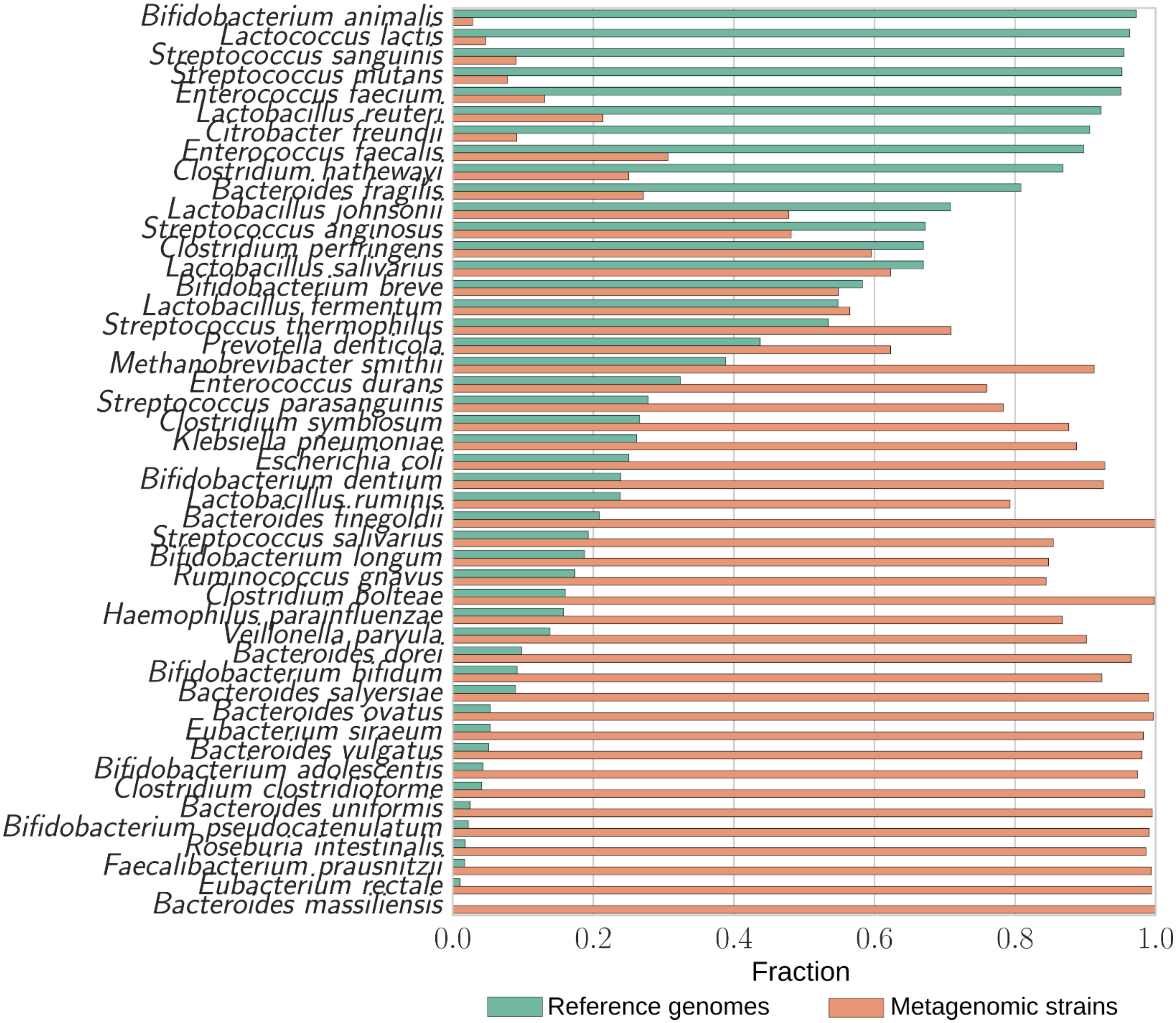

SUPPORTING REFERENCES

1. The Human Microbiome Consortium (2010) HMMC - Mock Community 16S & WGS Reads (http://hmpdacc.org/HMMC/).
2. Ren B, Truong DT, & Huttenhower C (2014) SynMetaP: A tool for simulating shotgun metagenomic sequencing data (https://bitbucket.org/Boyur/synmetap)).
3. Luo C*, et al.* (2015) ConStrains identifies microbial strains in metagenomic datasets. *Nat Biotechnol* 33(10):1045-1052.
4. Nielsen HB*, et al.* (2014) Identification and assembly of genomes and genetic elements in complex metagenomic samples without using reference genomes. *Nature biotechnology* 32(8):822-828.
5. Seemann T (2014) Prokka: rapid prokaryotic genome annotation. *Bioinformatics*:btu153.
6. Page AJ*, et al.* (2015) Roary: rapid large-scale prokaryote pan genome analysis. *Bioinformatics* 31(22):3691-3693.
7. Truong DT*, et al.* (2015) MetaPhlAn2 for enhanced metagenomic taxonomic profiling. *Nat Meth* 12(10):902-903.
8. Langmead B & Salzberg SL (2012) Fast gapped-read alignment with Bowtie 2. *Nature methods* 9(4):357-359.
9. Li H*, et al.* (2009) The sequence alignment/map format and SAMtools. *Bioinformatics (Oxford, England)* 25(16):2078-2079.
10. Altschul SF, Gish W, Miller W, Myers EW, & Lipman DJ (1990) Basic local alignment search tool. *Journal of molecular biology* 215(3):403-410.
11. Edgar RC (2004) MUSCLE: multiple sequence alignment with high accuracy and high throughput. *Nucleic acids research* 32(5):1792-1797.
12. Ott M, Zola J, Stamatakis A, & Aluru S (2007) Large-scale maximum likelihood-based phylogenetic analysis on the IBM BlueGene/L. *Proceedings of the 2007 ACM/IEEE conference on Supercomputing*, (ACM), p 4.
13. Heger A (2015) pysam: htslib interface for python, available from https://github.com/pysam-developers/pysam.
